# Ethanol-Mediated Novel Survival Strategy against Drought Stress in Plants

**DOI:** 10.1093/pcp/pcac114

**Published:** 2022-08-25

**Authors:** Khurram Bashir, Daisuke Todaka, Sultana Rasheed, Akihiro Matsui, Zarnab Ahmad, Kaori Sako, Yoshinori Utsumi, Anh Thu Vu, Maho Tanaka, Satoshi Takahashi, Junko Ishida, Yuuri Tsuboi, Shunsuke Watanabe, Yuri Kanno, Eigo Ando, Kwang-Chul Shin, Makoto Seito, Hinata Motegi, Muneo Sato, Rui Li, Saya Kikuchi, Miki Fujita, Miyako Kusano, Makoto Kobayashi, Yoshiki Habu, Atsushi J Nagano, Kanako Kawaura, Jun Kikuchi, Kazuki Saito, Masami Yokota Hirai, Mitsunori Seo, Kazuo Shinozaki, Toshinori Kinoshita, Motoaki Seki

**Affiliations:** Plant Genomic Network Research Team, RIKEN Center for Sustainable Resource Science, 1-7-22 Suehiro-cho, Tsurumi-ku, Yokohama, Kanagawa, 230-0045 Japan; Department of Life Sciences, SBA School of Science and Engineering, Lahore University of Management Sciences, DHA Phase 5, Lahore 54792, Pakistan; Plant Genomic Network Research Team, RIKEN Center for Sustainable Resource Science, 1-7-22 Suehiro-cho, Tsurumi-ku, Yokohama, Kanagawa, 230-0045 Japan; Plant Genomic Network Research Team, RIKEN Center for Sustainable Resource Science, 1-7-22 Suehiro-cho, Tsurumi-ku, Yokohama, Kanagawa, 230-0045 Japan; Plant Genomic Network Research Team, RIKEN Center for Sustainable Resource Science, 1-7-22 Suehiro-cho, Tsurumi-ku, Yokohama, Kanagawa, 230-0045 Japan; Plant Epigenome Regulation Laboratory, RIKEN Cluster for Pioneering Research, 2-1 Hirosawa, Wako, Saitama, 351-0198 Japan; Plant Genomic Network Research Team, RIKEN Center for Sustainable Resource Science, 1-7-22 Suehiro-cho, Tsurumi-ku, Yokohama, Kanagawa, 230-0045 Japan; Department of Life Sciences, SBA School of Science and Engineering, Lahore University of Management Sciences, DHA Phase 5, Lahore 54792, Pakistan; Plant Genomic Network Research Team, RIKEN Center for Sustainable Resource Science, 1-7-22 Suehiro-cho, Tsurumi-ku, Yokohama, Kanagawa, 230-0045 Japan; Department of Advanced Bioscience, Faculty of Agriculture, Kindai University, 3327-204 Nakamachi, Nara, 631-8505, Japan; Plant Genomic Network Research Team, RIKEN Center for Sustainable Resource Science, 1-7-22 Suehiro-cho, Tsurumi-ku, Yokohama, Kanagawa, 230-0045 Japan; Plant Genomic Network Research Team, RIKEN Center for Sustainable Resource Science, 1-7-22 Suehiro-cho, Tsurumi-ku, Yokohama, Kanagawa, 230-0045 Japan; Plant Genomic Network Research Team, RIKEN Center for Sustainable Resource Science, 1-7-22 Suehiro-cho, Tsurumi-ku, Yokohama, Kanagawa, 230-0045 Japan; Plant Epigenome Regulation Laboratory, RIKEN Cluster for Pioneering Research, 2-1 Hirosawa, Wako, Saitama, 351-0198 Japan; Plant Genomic Network Research Team, RIKEN Center for Sustainable Resource Science, 1-7-22 Suehiro-cho, Tsurumi-ku, Yokohama, Kanagawa, 230-0045 Japan; Plant Epigenome Regulation Laboratory, RIKEN Cluster for Pioneering Research, 2-1 Hirosawa, Wako, Saitama, 351-0198 Japan; Plant Genomic Network Research Team, RIKEN Center for Sustainable Resource Science, 1-7-22 Suehiro-cho, Tsurumi-ku, Yokohama, Kanagawa, 230-0045 Japan; Plant Epigenome Regulation Laboratory, RIKEN Cluster for Pioneering Research, 2-1 Hirosawa, Wako, Saitama, 351-0198 Japan; Environmental Metabolic Analysis Research Team, RIKEN Center for Sustainable Resource Science, 1-7-22 Suehiro-cho, Tsurumi-ku, Yokohama, Kanagawa, 230-0045 Japan; Dormancy and Adaptation Research Unit, RIKEN Center for Sustainable Resource Science, 1-7-22 Suehiro-cho, Tsurumi-ku, Yokohama, Kanagawa, 230-0045, Japan; IPSiM, University of Montpellier, CNRS, INRAE, Institut Agro, Montpellier 34060, France; Dormancy and Adaptation Research Unit, RIKEN Center for Sustainable Resource Science, 1-7-22 Suehiro-cho, Tsurumi-ku, Yokohama, Kanagawa, 230-0045, Japan; Division of Biological Sciences, Graduate School of Science, Nagoya University, Chikusa, Nagoya, 464-8602 Japan; Department of Biological Sciences, School of Science, The University of Tokyo, 7-3-1 Hongo, Bunkyo-ku, Tokyo, 113-0033, Japan; Division of Biological Sciences, Graduate School of Science, Nagoya University, Chikusa, Nagoya, 464-8602 Japan; Kihara Institute for Biological Research, Yokohama City University, 641-12 Maiokacho, Totsuka Ward, Yokohama, Kanagawa, 244-0813 Japan; Plant Genomic Network Research Team, RIKEN Center for Sustainable Resource Science, 1-7-22 Suehiro-cho, Tsurumi-ku, Yokohama, Kanagawa, 230-0045 Japan; Kihara Institute for Biological Research, Yokohama City University, 641-12 Maiokacho, Totsuka Ward, Yokohama, Kanagawa, 244-0813 Japan; Mass Spectrometry and Microscopy Unit, RIKEN Center for Sustainable Resource Science, 1-7-22 Suehiro-cho, Tsurumi-ku, Yokohama, Kanagawa, 230-0045 Japan; Metabolic Systems Research Team, RIKEN Center for Sustainable Resource Science, 1-7-22 Suehiro-cho, Tsurumi-ku, Yokohama, Kanagawa, 230-0045 Japan; Metabolic Systems Research Team, RIKEN Center for Sustainable Resource Science, 1-7-22 Suehiro-cho, Tsurumi-ku, Yokohama, Kanagawa, 230-0045 Japan; Gene Discovery Research Group, RIKEN Center for Sustainable Resource Science, 1-7-22 Suehiro-cho, Tsurumi-ku, Yokohama, Kanagawa, 230-0045 Japan; Mass Spectrometry and Microscopy Unit, RIKEN Center for Sustainable Resource Science, 1-7-22 Suehiro-cho, Tsurumi-ku, Yokohama, Kanagawa, 230-0045 Japan; Gene Discovery Research Group, RIKEN Center for Sustainable Resource Science, 1-7-22 Suehiro-cho, Tsurumi-ku, Yokohama, Kanagawa, 230-0045 Japan; Metabolomics Research Group, RIKEN Center for Sustainable Resource Science, 1-7-22 Suehiro-cho, Tsurumi-ku, Yokohama, Kanagawa 230-0045 Japan; Graduate School of Life and Environmental Science, University of Tsukuba, 1-1-1 Tennodai, Tsukuba, Ibaraki, 305-8572 Japan; Metabolomics Research Group, RIKEN Center for Sustainable Resource Science, 1-7-22 Suehiro-cho, Tsurumi-ku, Yokohama, Kanagawa 230-0045 Japan; Graduate School of Life and Environmental Science, University of Tsukuba, 1-1-1 Tennodai, Tsukuba, Ibaraki, 305-8572 Japan; Institute of Agrobiological Sciences, National Agriculture and Food Research Organization, 2-1-2 Kannondai, Tsukuba, Ibaraki, 305-8602 Japan; Faculty of Agriculture, Ryukoku University, Yokotani 1-5, Seta Oe-cho, Otsu, Shiga, 520-2914, Japan; Institute for Advanced Biosciences, Keio University, Tsuruoka, Yamagata, 997-0017 Japan; Kihara Institute for Biological Research, Yokohama City University, 641-12 Maiokacho, Totsuka Ward, Yokohama, Kanagawa, 244-0813 Japan; Environmental Metabolic Analysis Research Team, RIKEN Center for Sustainable Resource Science, 1-7-22 Suehiro-cho, Tsurumi-ku, Yokohama, Kanagawa, 230-0045 Japan; Graduate School of Medical Life Science, Yokohama City University, 1-7-22 Suehiro-cho, Tsurumi-ku, Yokohama, Kanagawa, 230-0045 Japan; Department of Applied Biosciences, Graduate School of Bioagricultural Sciences, Nagoya University, Chikusa, Nagoya, Aichi, 464-8601 Japan; Metabolomics Research Group, RIKEN Center for Sustainable Resource Science, 1-7-22 Suehiro-cho, Tsurumi-ku, Yokohama, Kanagawa 230-0045 Japan; Mass Spectrometry and Microscopy Unit, RIKEN Center for Sustainable Resource Science, 1-7-22 Suehiro-cho, Tsurumi-ku, Yokohama, Kanagawa, 230-0045 Japan; Department of Applied Biosciences, Graduate School of Bioagricultural Sciences, Nagoya University, Chikusa, Nagoya, Aichi, 464-8601 Japan; Metabolic Systems Research Team, RIKEN Center for Sustainable Resource Science, 1-7-22 Suehiro-cho, Tsurumi-ku, Yokohama, Kanagawa, 230-0045 Japan; Dormancy and Adaptation Research Unit, RIKEN Center for Sustainable Resource Science, 1-7-22 Suehiro-cho, Tsurumi-ku, Yokohama, Kanagawa, 230-0045, Japan; Gene Discovery Research Group, RIKEN Center for Sustainable Resource Science, 1-7-22 Suehiro-cho, Tsurumi-ku, Yokohama, Kanagawa, 230-0045 Japan; Division of Biological Sciences, Graduate School of Science, Nagoya University, Chikusa, Nagoya, 464-8602 Japan; Institute of Transformative Bio-Molecules (WPI-ITbM), Nagoya University, Chikusa, Nagoya, Aichi, 464-8601 Japan; Plant Genomic Network Research Team, RIKEN Center for Sustainable Resource Science, 1-7-22 Suehiro-cho, Tsurumi-ku, Yokohama, Kanagawa, 230-0045 Japan; Plant Epigenome Regulation Laboratory, RIKEN Cluster for Pioneering Research, 2-1 Hirosawa, Wako, Saitama, 351-0198 Japan; Kihara Institute for Biological Research, Yokohama City University, 641-12 Maiokacho, Totsuka Ward, Yokohama, Kanagawa, 244-0813 Japan

**Keywords:** ABA, Chemical priming, Drought tolerance, Ethanol, Gluconeogenesis, Stomatal closure

## Abstract

Water scarcity is a serious agricultural problem causing significant losses to crop yield and product quality. The development of technologies to mitigate the damage caused by drought stress is essential for ensuring a sustainable food supply for the increasing global population. We herein report that the exogenous application of ethanol, an inexpensive and environmentally friendly chemical, significantly enhances drought tolerance in *Arabidopsis thaliana*, rice and wheat. The transcriptomic analyses of ethanol-treated plants revealed the upregulation of genes related to sucrose and starch metabolism, phenylpropanoids and glucosinolate biosynthesis, while metabolomic analysis showed an increased accumulation of sugars, glucosinolates and drought-tolerance-related amino acids. The phenotyping analysis indicated that drought-induced water loss was delayed in the ethanol-treated plants. Furthermore, ethanol treatment induced stomatal closure, resulting in decreased transpiration rate and increased leaf water contents under drought stress conditions. The ethanol treatment did not enhance drought tolerance in the mutant of *ABI1*, a negative regulator of abscisic acid (ABA) signaling in Arabidopsis, indicating that ABA signaling contributes to ethanol-mediated drought tolerance. The nuclear magnetic resonance analysis using ^13^C-labeled ethanol indicated that gluconeogenesis is involved in the accumulation of sugars. The ethanol treatment did not enhance the drought tolerance in the aldehyde dehydrogenase (*aldh*) triple mutant (*aldh2b4/aldh2b7/aldh2c4*). These results show that ABA signaling and acetic acid biosynthesis are involved in ethanol-mediated drought tolerance and that chemical priming through ethanol application regulates sugar accumulation and gluconeogenesis, leading to enhanced drought tolerance and sustained plant growth. These findings highlight a new survival strategy for increasing crop production under water-limited conditions.

## Introduction

A lack of available water limits plant growth and photosynthetic capabilities, resulting in decreased crop yield. The global population has been increasing rapidly and is projected to reach ∼9.5 billion by 2050. Hence, ensuring that sufficient food continues to be produced for the increasing population is a major challenge for plant scientists (Hickey et al. 2019). Meanwhile, environmental stresses are worsening, including drought, which is expected to increase in frequency and duration (Ault 2020). Achieving food security while promoting sustainable agriculture is included in the United Nations’ Sustainable Development Goals (Goal 2: Zero Hunger; Kroll 2015). A thorough understanding of the plant drought stress response is essential for developing effective strategies to mitigate the adverse effects of drought stress (Gupta et al. 2020, Yoshida et al. 2021, Kuromori et al. 2022, Zhang et al. 2022). In plants, stomata regulate gaseous exchange and are integral to regulating water and photosynthesis (Hsu et al. 2021). Several approaches involving conventional and transgenic technologies have been used to improve crop growth under water-limited conditions, including the regulation of transcription factors, osmolyte biosynthesis, peptides and metabolic enzymes (Kim et al. 2017, Takahashi et al. 2018, Vaidya et al. 2019, Ahmad et al. 2021). Much of the related research has focused on decreasing the transpiration rate under drought conditions by regulating stomatal opening (Hetherington and Woodward 2003). The exogenous application of plant metabolites reportedly enhances plant stress tolerance (Sako et al. 2021). Acetic acid increases drought tolerance, while ethanol can mitigate the detrimental effects of salinity and heat stress (Kim et al. 2017, Nguyen et al. 2017, Ogawa et al. 2021, Sako et al. 2021, Matsui et al. 2022). In plants, ethanol fermentation ensures that energy is conserved under stress conditions (Tadege et al. 1999). In the ethanol fermentation pathway, pyruvate is decarboxylated to generate acetaldehyde by pyruvate decarboxylase, which is specifically conserved in plants, mosses and fungi (Kim et al. 2017). Acetaldehyde is then reduced to ethanol via a reaction catalyzed by alcohol dehydrogenase (ADH), which is accompanied by the oxidation of nicotinamide adenine dinucleotide to NAD^+^ (**Supplementary Fig. S1**). This reaction, which is reversible, enables ethanol to be readily transformed into acetic acid through the acetaldehyde intermediate by aldehyde dehydrogenase (ALDH) enzymes (Tadege et al. 1999, Rasheed et al. 2018). The resulting acetic acid compound can be metabolized through the tricarboxylic acid (TCA) or glyoxylate cycle (Tadege et al. 1999, Fu et al. 2020).

Chemical priming offers several key advantages over transgenic technology, but it must be affordable for farmers, environmentally friendly and safe for humans, animals and plants. In this study, we demonstrate that chemical priming with ethanol, an inexpensive and environmentally friendly chemical, significantly enhances the drought tolerance in *Arabidopsis thaliana*, rice and wheat. Ethanol-mediated drought tolerance may be exploited to mitigate the adverse effects of drought stress and represents an alternative to the development of transgenic plants or laborious classical breeding methods.

## Results

### Enhanced plant drought tolerance through the regulation of stomatal closure by ethanol treatment

**Fig. 1:**
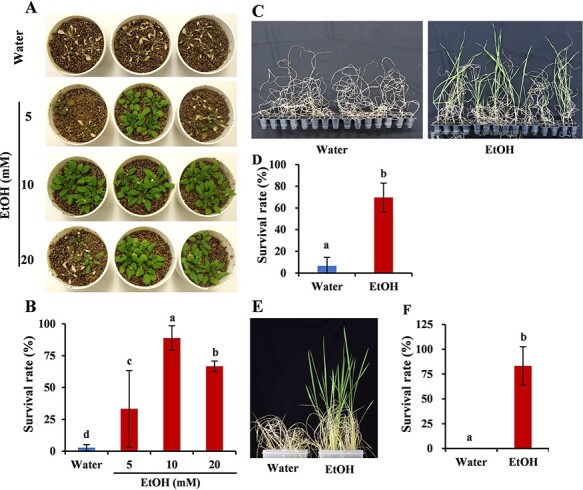
Ethanol enhances plant drought tolerance. (A, B) Different ethanol (EtOH) concentrations enhanced the drought tolerance of *A. thaliana*. Data were recorded 3 d after plants were rewatered. (C, D) 50 mM ethanol treatment significantly enhanced the drought tolerance of wheat (*T. aestivum*, Chinese Spring). Data were recorded 30 d after plants were rewatered. (E, F) 100 mM ethanol treatment significantly enhanced the drought tolerance of rice (*O. sativa* L., Nipponbare). Data were recorded 20 d after plants were rewatered. Different letters indicate significant differences according to the Tukey–Kramer test (*P* < 0.05); (A–D) *n* = 3 and (E, F) *n* = 4.

Previous research revealed that the expression of *ADH1* increases under drought conditions (Rasheed et al. 2016). *ADH1* converts acetaldehyde to ethanol in a reversible manner (Bui et al. 2019), suggesting that the exogenous application of ethanol might enhance plant drought tolerance. First, we examined the effect of exogenous ethanol on plants by treating *A. thaliana* roots with 5, 10 and 20 mM ethanol for 3 d. All three concentrations enhanced drought tolerance; however, the effect of 10 mM ethanol was greater than that of 5 and 20 mM ethanol (**Fig. 1A**, **B**). Thus, 10 mM ethanol was used for most of the subsequent analyses of *A. thaliana*. Ethanol treatments also improved the drought tolerance in wheat (50 mM; **Fig. 1C**, **D**) and rice (100 mM; **Fig. 1E**, **F**). These results demonstrate that ethanol can decrease drought-induced damages to monocots and dicots.

**Fig. 2:**
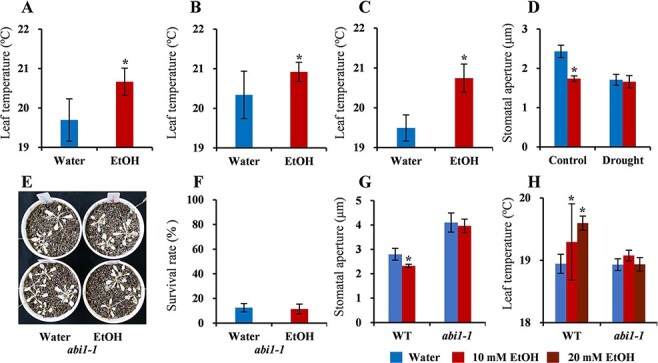
ABA signaling is involved in ethanol-mediated drought tolerance. The roots of 19-day-old WT plants were treated with 10 mM ethanol for 3 d and then subjected to drought stress. (A) Leaf temperature at 72 h after the ethanol treatment (0 DAD). (B) 1 DAD. (C) 3 DAD. (D) Stomatal aperture under 7-d watered control conditions and at 7 DAD. Asterisks indicate significant differences (compared with the control conditions) according to the Student-Newman-Keuls (SNK) test (*P* < 0.05); (A–C) *n* > 20 and (D) *n* = 4. (E, F) Drought test of water- and ethanol-treated *abi1-1* plants. (G) Stomatal aperture of WT (L*er*) and the *abi1-1* mutant at 1-day after 3-day ethanol treatment. (H) Leaf temperature measurement of WT (L*er*) and the *abi1-1* mutant at 3-d ethanol treatment. There were no significant differences between the control and ethanol-treated *abi1-1* plants according to the SNK test (*P* < 0.05); *n* = 3.

In ethanol-treated plants, the leaf temperature increased at 72 h after the ethanol treatment [0 d after drought (DAD)] and at 1 and 3 DAD (**Fig. 2A****–C**). The stomatal aperture decreased in the ethanol-treated plants under control conditions, but this effect of ethanol decreased at 7 DAD (**Fig. 2D**). Although we did not measure the stomatal aperture at 3 DAD, the observed increase in leaf temperature at this time point was indicative of stomatal closure and a decreased transpiration rate during the early response to drought stress. An analysis of the abscisic acid (ABA) contents in the roots and shoots of ethanol- and water-treated plants at several time points revealed a lack of significant differences between the two treatments (**Supplementary Fig. S2**). To further investigate the mechanism regulating stomatal closure, we tested if ethanol enhances drought tolerance in an ABA signaling pathway mutant, *ABA insensitive 1-1* (*abi1-1*) (Gosti et al. 1999). Ethanol treatment did not enhance drought tolerance in the *abi1-1* mutant plants (**Fig. 2E**, **F**), although enhanced drought tolerance was observed in *A. thaliana* Landsberg *erecta* ecotype (**Supplementary Fig. S3**). Ethanol treatment also neither decreased the stomatal aperture nor increased the leaf temperature in the *abi1-1* mutant (**Fig. 2G**, **H**). These results indicate that ABA signaling is involved in ethanol-mediated drought tolerance.

### Identification of the genes regulated by ethanol treatment

A plant growth system using ceramic-based granular soil can be used to isolate roots and shoots separately under drought conditions (Rasheed et al. 2016). In the current study, ethanol-mediated drought tolerance was observed in plants grown in ceramic-based granular soil (**Supplementary Fig. S4**). For the microarray analysis, the roots and shoots were harvested separately at 0, 3, 24 and 72 h after the ethanol treatment and at 1, 3 and 5 DAD. The microarray analysis of the roots revealed the upregulated expression of 31, 150 and 26 genes at 3, 24 and 72 h after the ethanol treatment, respectively (**Supplementary Fig. S5A**). During the drought treatment, the expression levels of 86, 82 and 106 genes were upregulated at 1, 3 and 5 DAD, respectively (**Supplementary Fig. S5B**). In the shoots, the expression levels of 34, 95 and 30 genes were upregulated at 3, 24 and 72 h after the ethanol treatment, respectively (**Supplementary Fig. S5C**). Moreover, 312, 350 and 755 genes had upregulated expression levels in the shoots at 1, 3 and 5 DAD, respectively (**Supplementary Fig. S5D**). The *Nine-Cis-Epoxycarotenoid Dioxygenase 3* expression level increased at 24 h after the ethanol treatment and at 3 DAD in the roots and at 1 and 3 DAD in the shoots (**Supplementary Tables S1 and S2**). The expression levels of several other drought-tolerance-related genes belonging to the ABA-dependent pathway (*MYC2, Abscisic Acid-Responsive Element Binding Protein1, delta(1)-Pyrroline-5-Carboxylate Synthetase1 and Responsive to Desiccation 29B*) and the ABA-independent pathway (*Dehydration Responsive Element-Binding Protein 1A and Galactinol Synthase 3*) were also upregulated in the shoots by the ethanol and drought treatments (**Supplementary Tables S1 and S2**,   **Supplementary Fig. S6**). The expression levels of the genes involved in the biosynthesis of aliphatic glucosinolates, which contribute to drought tolerance (Salehin et al. 2019), were higher in the ethanol-pretreated shoots during the drought treatment (**Supplementary Tables S1 and S3**). Genes related to flavonoid/anthocyanin biosynthesis were also upregulated (**Supplementary Table S1**). The Gene Ontology (GO) enrichment analysis indicated that the upregulated genes in the shoots were related to glucosinolates and responses to jasmonate (JA) (**Supplementary Table S4**). The metabolic accumulation of several glucosinolates was greater in the ethanol-treated shoots than in the water-treated shoots (**Supplementary Table S5**).

To further elucidate the transcriptomic changes associated with the later stages of drought stress, RNA-sequencing (RNA-seq) analyses were performed twice. The first RNA-seq analysis was performed to analyze *A. thaliana* shoots at 0, 1, 3, 5 and 7 DAD. The second RNA-seq analysis was performed to analyze the effects of slight changes in growth conditions at 0, 10 and 13 DAD. There was a considerable overlap in the transcriptomic changes at 7 and 13 DAD (**Supplementary Fig. S7A and B**, **Supplementary Table S6**). RNA-seq data were validated through quantitative Reverse Transcription PCR (qRT-PCR) analysis (**Supplementary Fig. S8)**. The enriched GO terms among the upregulated genes in the shoots of the ethanol-treated and drought-stressed plants included glucosinolate biosynthesis, NAD(P)H dehydrogenase complex assembly and cellular oxidant detoxification (**Supplementary Table S7**). About 600 genes had lower expression levels in the ethanol-treated plants than in the water-treated plants (**Supplementary Table S8**).

### Delay of drought-induced water loss by ethanol treatment

**Fig. 3:**
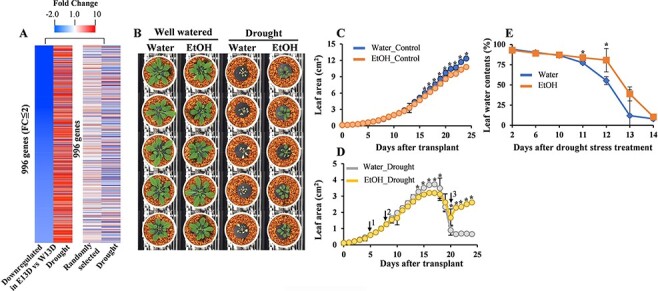
Effect of the ethanol treatment on plant growth and survival potential under drought conditions. (A) Heatmap analysis of downregulated genes at 13 DAD from RNA-seq data. *A. thaliana* genes were randomly selected; Log_2_ (fold change) ≤ −1 and FDR < 0.1. (B–E) 2-week-old *A. thaliana* plants were treated with 10 mM ethanol or water for 3 d and then subjected to drought stress. (B) Plants grown under well-watered and drought conditions. (C–E) Leaf area (C, D) and leaf water content (E) of the plants grown under water-limited conditions were recorded using the RIPPS (Fujita et al. 2018). Asterisks indicate significant differences among the water- and ethanol-treated plants according to the SNK test (*P* < 0.05); *n* = 5. (D) Arrows indicate the initiation of the ethanol treatment^1^, drought treatment^2^ and rewatering^3^.

**Fig. 4:**
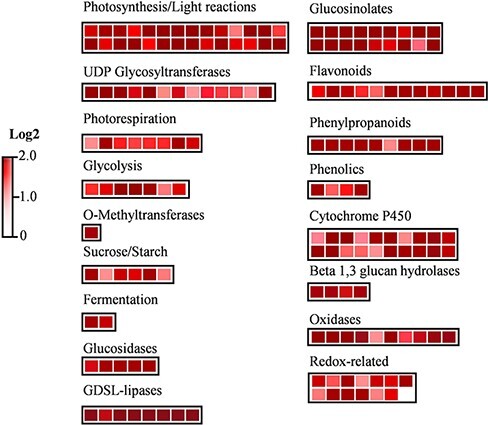
Summary of the MapMan analysis. Genes upregulated in the first (7 DAD) and second (13 DAD) RNA-seq analyses were plotted using MapMan 3.6.0RC1. Redox-related genes include those associated with thioredoxin, ascorbate/glutathione and dismutase/catalase. Oxidase-related genes include those encoding oxidoreductase, dioxygenase, flavin-monooxygenase and glucosinolate S-oxygenase. *n* = 3, fold change ≥ 2 and FDR < 0.1.

RNA-seq analysis revealed that the drought-inducible genes were expressed at lower levels in the plants pretreated with ethanol than in the water-treated control plants at later stages (7 and 13 DAD) of drought treatment (**Fig. 3A**, **Supplementary Fig. S7C, D**). Ethanol-mediated drought tolerance was further monitored by conducting a RIKEN Integrated Plant Phenotyping System (RIPPS) analysis (Fujita et al. 2018). The ethanol treatment enhanced the drought tolerance of *A. thaliana* plants grown in the RIPPS facility (**Fig. 3B**, Bashir et al. **Supplementary Video 1**). Under well-watered conditions, compared with the water-treated plants, the growth of the ethanol-treated plants was slightly inhibited, as indicated by the leaf area measurements (**Fig. 3B**, **C**). Under drought conditions, plant growth was similar until 5 DAD, after which the ethanol-treated plants were slightly smaller than the water-treated plants from 6 to 10 DAD. From 11 DAD onward, the ethanol-treated plants grew better than the water-treated plants (**Fig. 3D**). At 11 and 12 DAD, the shoot water contents were significantly higher for the ethanol-treated plants than for the water-treated plants (**Fig. 3E**). At this stage, the RNA-seq analysis revealed the upregulated expression of several genes related to photosynthesis (light reactions), photorespiration, sucrose and starch metabolism and the biosynthesis of glucosinolates, flavonoids, phenylpropanoids and phenolics and redox-related genes, as classified by the MapMan analysis (**Fig. 4**, **Supplementary Table S9**). MapMan is a data visualization tool that presents large datasets such as microarray or RNA-seq data as diagrams showing metabolic pathways or other processes based on their expression patterns (Schwacke et al. 2019).

### Involvement of gluconeogenesis and acetic acid biosynthesis in ethanol-mediated drought tolerance

**Fig. 5:**
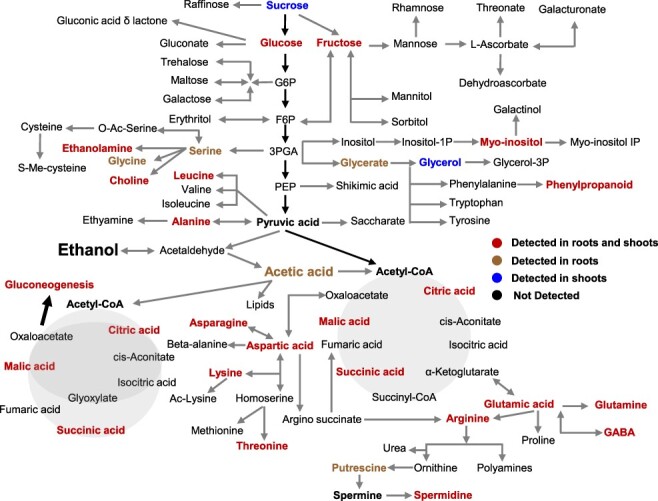
Summary of the NMR analysis of ^13^C-labeled ethanol-treated Arabidopsis roots and shoots. Two-week-old *A. thaliana* plants were treated with 10 mM ^13^C-labeled ethanol. Root and shoot tissue samples were collected at 24- and 72-h post-ethanol treatment and at 1, 3 and 5 DAD. ^13^C-labeled metabolites detected through HSQC-NMR analysis at all time points are shown in a tissue-specific manner. Metabolites shown in red were detected in both roots and shoots and those shown in brown were detected only in roots, while those in blue were detected in shoots only. Other metabolites shown in black were not detected in this analysis.

For the nuclear magnetic resonance (NMR) analysis, 10 mM ^13^C-labeled ethanol was fed to the plants through the roots. The roots and shoots were collected separately at 24 and 72 h after the ethanol treatment. Plants were then subjected to drought conditions, with samples collected at 1, 3 and 5 DAD. At 24 h after the ethanol treatment, the accumulation of several ^13^C-labeled metabolites indicated that ethanol had been taken up by the plants and incorporated into the metabolites in the root and shoot tissues (**Fig. 5**, **Supplementary Figs. S9, S10**). Labeled glucose, fructose, sucrose and several other metabolites synthesized from these sugars, such as glycerate, glycerol and myoinositol, were also detected (**Supplementary Figs. S9, S10**). These results reflect an immediate conversion of ethanol into sugars through gluconeogenesis (**Fig. 5**). A 2D *J-*resolved NMR analysis of the roots and shoots confirmed the accumulation of several metabolites in response to the drought treatment (**Supplementary Figs. S11, S12**). The upregulated expression of the peroxisomal malate dehydrogenase gene (At5g09660) (Cousins et al. 2008) (**Supplementary Table S6**) provided further evidence of the involvement of gluconeogenesis.

**Fig. 6:**
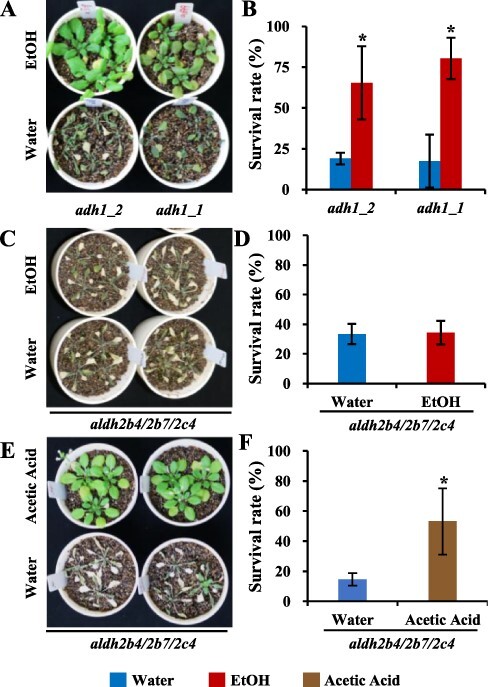
Ethanol enhances the drought tolerance of the *adh1* mutants, but not the *aldh* triple mutant. Two-week-old *A. thaliana adh1* and *aldh* triple mutant (*aldh2b4/aldh2b7/aldh2c4*) plants were treated with 10 mM ethanol for 3 d and then subjected to drought stress. (A, C) The *adh1* (A) and *aldh* triple mutant (C) plants were photographed at 4 d after rewatering. (B, D) Survival rate (%) of the *adh1* (B) and *aldh* triple mutant (D) plants. (E, F). Two-week-old *aldh* triple mutant plants were treated with 10 mM acetic acid for 3 d and then subjected to drought stress treatment. (E) Plants were photographed at 4 d after rewatering. (F) Survival rate (%) of the *aldh* triple mutant plants. Asterisks indicate significant differences (compared with the control) according to the SNK test (*P* < 0.05; *n* = 3).

We then tested whether the ethanol treatment enhanced the drought tolerance of the *adh1* mutants. Following the ethanol treatment, the survival rate was higher for the T-DNA insertional mutants *adh1-1* and *adh1-2* than for the water-treated mutant plants (**Fig. 6A**, **B**). We further tested if the conversion of ethanol into acetic acid contributes to drought stress tolerance in ethanol-treated plants. In *A. thaliana*, three functional homologs *ALDH2B4, ALDH2B7* and *ALDH2C4* are likely involved in the conversion of acetaldehyde into acetic acid (Wei et al. 2009). The ethanol treatment did not enhance the drought tolerance in the *ALDH* triple mutant (*aldh2b4/aldh2b7/aldh2c4*), suggesting that the conversion of ethanol into acetic acid might be critical for the ethanol-mediated drought tolerance in *A. thaliana* (**Fig. 6C**, **D**). On the other hand, enhanced drought tolerance was observed in the *aldh* triple mutant plants treated with acetic acid (**Fig. 6E**, **F**).

Acetic acid considerably hinders seed germination and plant growth in *A. thaliana* at concentrations as low as 1 mM (**Supplementary Fig. S13**), whereas ethanol does not affect seed germination and plant growth in *A. thaliana* up to the concentrations of 15 mM (Matsui et al. 2022). We also tested if the prolonged application of ethanol and acetic acid affects plant growth. For this purpose, 15-day-old *A. thaliana* plants were treated with 10 mM ethanol or acetic acid for five continuous weeks by renewing the ethanol and acetic acid solutions on a weekly basis. The prolonged application of both ethanol and acetic acid affected plant growth; however, the growth retardation by the acetic acid application was more severe than the ethanol application (**Supplementary Fig. S14**). We further analyzed the leaf temperatures of ethanol- and acetic-acid-treated plants to test if the acetic acid treatment induces stomatal closure. Ethanol treatment increased the leaf temperature, while acetic acid treatment did not (**Supplementary Fig. S15**), highlighting the difference between ethanol- and acetic-acid-treatment-mediated drought tolerance mechanisms. Furthermore, we tested if the JA signaling is involved in the ethanol-mediated drought tolerance in Arabidopsis. Coronatine-insensitive 1 (COI1) is a JA receptor regulating JA response in Arabidopsis, and plants lacking functional COI1 protein (*coi1*-16B) are sensitive to drought stress (Thines et al. 2007, Noir et al. 2013, Kim et al. 2017). We tested the effect of ethanol treatment on the growth and drought tolerance of the *coi1*-16B mutant plants. Ethanol-mediated response was similar between Wild type (WT) and *coi1*-16B plants in terms of plant growth under drought stress (**Supplementary Fig. S16**). The *coi1*-16B plants were more sensitive to drought as compared to the WT plants, and ethanol treatment enhanced drought tolerance in both WT and *coi1*-16B plants (**Supplementary Fig. S17**).

The reprogramming of the plant metabolome in the ethanol-treated roots and shoots has been indicated by gas chromatography–mass spectrometry (GC–MS) analysis (**Supplementary Tables S10 and S11**). The root metabolome of the ethanol-treated and water-treated plants was similar before the initiation of the drought treatment, but differences were detected under drought conditions. A substantial increase in the accumulation of several drought-tolerance-related amino acids, including proline, leucine, valine and glutamine, as well as sugars, such as fructose, glucose 6-phosphate, sucrose and maltose, was observed in the ethanol-treated roots (**Supplementary Table S10**), and shoots (**Supplementary Table S11**).

## Discussion

Chemical priming via the exogenous application of cost-effective and environmentally friendly metabolites may offer several advantages over classical breeding and transgenic technology in plants (Sako et al. 2021). In the current study, we demonstrate that ethanol treatment enhances drought tolerance in plants, such as Arabidopsis, rice and wheat. ABA signaling and acetic acid biosynthesis are involved in ethanol-mediated drought tolerance. Chemical priming through ethanol application induces the accumulation of sugars through gluconeogenesis, glucosinolates, and drought tolerance-related amino acids, leading to enhanced drought tolerance in plants. The ethanol-treated plants conserve water by closing their stomata, as indicated by their increased leaf temperature, decreased stomatal aperture and increased water contents (**Figs. 2A**–**D**, **3E**). Accordingly, the ethanol-treated plants are better prepared to withstand drought stress as compared to the control plants.

**Fig. 7:**
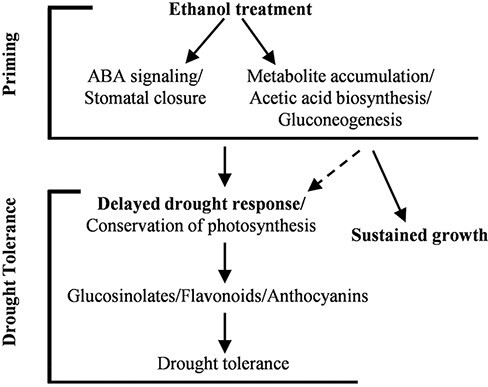
The proposed mechanism underlying ethanol-mediated plant drought tolerance. The ethanol treatment primes the cellular and metabolic environment to enhance drought tolerance in plants.

Under drought conditions, ABA induces stomatal closure and regulates the expression of several stress-responsive genes. The ABA signaling pathway is essential for the regulation of plant drought stress responses (Kuromori et al. 2022). A previous study revealed that ABI1 negatively regulates ABA signaling in plants (Gosti et al. 1999). Ethanol-mediated drought tolerance was not observed in the *abi1-1* mutant (**Fig. 2E**, **F**), implying that ABA signaling contributes to ethanol-mediated drought tolerance. There were no significant differences in the accumulation of ABA in the roots and shoots between the ethanol-treated and water-treated WT plants at any stage before or after the initiation of the drought treatment (**Supplementary Fig. S2**). This observation may be explained by the following: (i) the expression of a few genes related to ABA biosynthesis and catabolism was upregulated in the ethanol-treated plants (**Supplementary Tables S1 and S2**), resulting in rapid ABA metabolic changes; (ii) the whole shoots and roots were used for ABA measurements, and the increased ABA accumulation might occur in specific cells (Kuromori et al. 2022) and (iii) unknown mechanisms might be involved in the ethanol-mediated drought tolerance in plants. A group of ethylene-receptor-related sensor histidine kinases is essential for ABA and osmostress responses in the moss *Physcomitrium patens* (Toriyama et al. 2022), and a similar mechanism might contribute to the ethanol-mediated drought tolerance in plants. In addition to ABA signaling, the exogenous ethanol treatment could enhance drought tolerance via drought acclimation by reprograming the metabolomic and transcriptomic profiles that regulate the water conservation within the plants, leading to sustained photosynthetic activity, accumulation of glucosinolates/flavonoids/anthocyanins and gluconeogenesis (**Fig. 7**).

The upregulated expression of drought-tolerance-related genes in the ethanol-treated plants indicates that these plants might be better prepared for the perception of drought stress than the control plants (**Supplementary Table S1**). Additionally, an increased expression of genes related to glucosinolate biosynthesis, sucrose and starch metabolism, phenolics and the cytochrome P450 family may also be associated with plant drought tolerance. Glucosinolates play an important role in drought stress tolerance, and their nutritional effects in humans as well as their metabolic pathways in plants have been well characterized (Sønderby et al. 2010, Salehin et al. 2019, Sugiyama et al. 2021). Ethanol-mediated drought tolerance might be related to the increased accumulation of glucosinolates in ethanol-treated shoots at 7 DAD (**Supplementary Table S5**). The upregulated expression of genes participating in the detoxification of reactive oxygen species (ROS) was also observed (**Fig. 4**, **Supplementary Table S1**). The ethanol-treatment-mediated ROS detoxification enhances salt stress tolerance in Arabidopsis (Nguyen et al. 2017).

In ethanol-treated plants, there seem to be two phases of the drought stress response. During the early phase of the drought stress response, the ethanol-treated plants conserved water by closing their stomata coupled with the upregulation of drought-inducible genes (**Fig. 2B**-**D**, **Supplementary Table S1**). As discussed earlier, ABA signaling might be involved in stomatal closure and ethanol-mediated drought tolerance. Hence, in ethanol-treated plants, ABA signaling and the regulation of drought-responsive genes might govern the ethanol-mediated drought stress response in the early phases of drought stress (**Fig. 7**). In the later stages of drought stress, no differences were observed in the stomatal aperture of ethanol- and water-treated plants (**Fig. 2D**), and the ethanol-treated plants showed higher leaf water contents and downregulation of drought-inducible genes as compared to the water-treated drought-stressed plants (**Fig. 3A**, **E**, **Supplementary Figs. S7, S8**). The upregulation of genes related to photosynthesis/light reactions and glycolysis (**Fig. 4**) in the ethanol-treated plants as compared to the water-treated plants during the later stages of drought stress indicates that photosynthetic activity and plant growth might be retained in the ethanol-treated drought-stressed plants (**Figs. 4**, **7**).

Ethanol can easily be converted into acetic acid, which is then incorporated into the TCA cycle, as indicated by the detection of ^13^C-labeled citrate, succinate and malate during the NMR analysis of the root and shoot tissues (**Fig. 5**, **Supplementary Figs. S9, S10**). Ethanol-mediated drought tolerance was observed in the *adh1* mutants (**Fig. 6A**, **B**). Only one functional copy of the *ADH* gene (*ADH1*) has been identified in *A. thaliana* (Bui et al. 2019). In other organisms, catalases and cytochrome P450 family members convert alcohol to acetaldehyde (Wilson and Matschinsky 2020). A similar mechanism might exist in Arabidopsis. Furthermore, in *A. thaliana*, ALDH2B4, ALDH2B7 and ALDH2C4 are likely involved in the conversion of acetaldehyde into acetic acid (Wei et al. 2009). Ethanol-mediated drought tolerance was not observed in the *aldh* triple mutant (*aldh2b4/aldh2b7/aldh2c4*; **Fig. 6C**, **D**). These results show that the conversion of ethanol into acetic acid is critical for ethanol-mediated drought tolerance. Acetic-acid-mediated drought tolerance has been previously reported in Arabidopsis and other plant species (Kim et al. 2017, Rasheed et al. 2018, Utsumi et al. 2019, Ogawa et al. 2021). Acetic acid triggers the JA signaling pathway to confer drought tolerance in Arabidopsis (Kim et al. 2017). The GO enrichment analysis revealed that the enrichment of genes related to JA responses in the ethanol-treated drought-stressed plants at 3 DAD (**Supplementary Table S4**), while such enrichment was not observed at later time points of drought-stressed plants. These results indicate that JA signaling pathway might be differently regulated in ethanol and acetic acid treatments. The acetic acid treatment hinders seed germination and plant growth on Murashige and Skoog (MS) medium as compared to the ethanol treatment (**Supplementary Fig. S13**; Matsui et al. 2022). Moreover, in soil a prolonged application of acetic acid considerably decreased the plant growth as compared to the ethanol-treated plants (**Supplementary Fig. S14**). Acetic acid treatment does not increase the leaf temperature, indicating that the stomatal closure might not be a major contributing factor to acetic-acid-mediated drought tolerance in Arabidopsis (**Supplementary Fig. S15**). COI regulates JA signaling in Arabidopsis (Thines et al. 2007), and the ethanol-treated *coi1*-16B mutant plants showed an increased survival rate as compared to the water-treated plants (**Supplementary Fig. S17**). This suggests that unlike acetic acid, the contribution of JA signaling is low for ethanol-mediated drought tolerance. These results indicate that despite the metabolic conversion of ethanol into acetic acid, ethanol-mediated drought tolerance exhibits several differences as compared to the acetic-acid-mediated drought tolerance.

The biosynthesis of several amino acids and metabolites is regulated by the TCA cycle (Michaeli et al. 2011). Hence, the metabolic conversion of ethanol to asparagine, aspartate, γ-aminobutyrate, glutamate, glutamine and lysine may be explained in this context (**Fig. 5**). In this study, ^13^C-labeled glucose, fructose, sucrose and several key metabolites synthesized from these sugars, such as glycerate, glycerol and myoinositol, were detected in the roots and shoots (**Fig. 5**, **Supplementary Figs. S9–S12**). These results indicate that labeled ethanol was readily converted into sugars through gluconeogenesis. The upregulated expression of glucose and starch metabolism-related genes and the accumulation of proline, leucine, valine and glutamine reflect the importance of sugar accumulation and/or gluconeogenesis for plant drought tolerance or sustained plant growth despite stomatal closure. The accumulation of metabolites and osmolytes during the early drought stress stage might have enabled the ethanol-treated plants to better withstand the harmful effects of drought stress than the control plants.

Gluconeogenesis is a fundamental metabolic process that allows organisms to make sugars from non-carbohydrate sources, including lipids and proteins (Graham 2008). The expression of genes encoding enzymes related to the glyoxylate cycle, such as isocitrate lyase and malate synthase, increases in response to drought stress in rice, but not in Arabidopsis (Maruyama et al. 2014). This indicates that the regulation of the glyoxylate cycle may be involved in the glucose accumulation induced by dehydration in rice, but not in Arabidopsis (Maruyama et al. 2014). The incorporation of ^13^C-labeled ethanol into sugars provides direct evidence that gluconeogenesis might be occurring in Arabidopsis under drought conditions. This may contribute to the accumulation of sugars and osmolytes, which ultimately leads to greater protection from drought stress. The incorporation of ^13^C-labeled ethanol into sugars was observed before the onset of drought stress, and it continued as the drought stress progressed (**Fig. 5**, **Supplementary Figs. S9, S10**). In the ethanol-treated plants, the upregulated expression of the peroxisomal malate dehydrogenase gene (Cousins et al. 2008) also indicates that gluconeogenesis may help mediate drought tolerance. Notably, gluconeogenesis is initiated by the degradation of lipids, such as triacylglycerols, to acetate, which is converted to acetyl-coenzyme A and then recycled back into sugars. Plant vegetative tissues do not accumulate a significant amount of triacylglycerols (Xu and Shanklin 2016). Because vegetative tissues do not have lipid stores, the external application of ethanol may be an efficient way to conserve energy in plants. Gluconeogenesis produces energy by converting ethanol to sugars and may compensate for the decreased growth potential caused by stomatal closure. Growth retardation has been observed in mutants and transgenic plants exhibiting drought tolerance (Claeys and Inzé 2013). The conversion of ethanol to sugars through gluconeogenesis might be a factor for maintaining plant growth, despite clear evidence that stomata were closed in the ethanol-treated plants (**Figs. 2**, **3**).

The development of new technologies to increase crop production under water-limited conditions will likely increase sustainable crop production under rapidly changing environmental conditions. Our results indicate that an exogenous ethanol treatment can increase drought tolerance via drought acclimation by reprograming the metabolomic and transcriptomic profiles that regulate the drought stress response (**Fig. 7**). Ethanol treatment can also enhance the drought tolerance in commercially important crops (rice and wheat), offering a unique opportunity for drought stress mitigation without the need for genetically modifying crop plants.

## Materials and Methods

### Plant materials and growth conditions

*Arabidopsis thaliana* (Col-0 ecotype) seeds were sown in Dio propagation mix no. 2 soil (DIO Chemicals, Tokyo, Japan). After a 3-day vernalization at 4°C in darkness, the seedlings were transferred to a greenhouse and grown at 22°C with a 16-h light (∼100 µmol m^−2^ s^−1^ photon flux density)/8-h dark cycle. The 17-day-old plants were treated with different ethanol concentrations (5, 10 and 20 mM) for 3 d. Ethanol treatment was initiated by replacing the water supply with ethanol solutions from the downside of the pots containing the plantlets. Plants were then subjected to drought stress by ceasing the water supply and removing excess water. For each treatment, six pots containing four plants each, harboring 24 plants in total, were used with three experimental replications (a total set of 72 plants per treatment). The *abscisic acid insensitive 1-1* (*abi1-1*), *coi1*-16B, *adh1-1* (SALK_052699; T-DNA insertional mutant) and *adh1-2* (SAIL_603_A05; T-DNA insertional mutant) single mutants, as well as the *ALDH* triple mutant (*aldh2b4/aldh2b7/aldh2c4*; CS69951), were grown in Dio propagation mix no. 2 soil and treated with ethanol as described earlier. The *aldh* triple mutant plants were treated with acetic acid and then subjected to drought stress as described previously (Kim et al. 2017).

For the transcriptome analyses involving microarray and RNA-seq methods (**Supplementary Fig. S18**) and the metabolome analysis using GC–MS, Liquid chromatography-tandem mass spectrometry (LC MS/MS) and NMR systems, *A. thaliana* seeds were sown, and the resulting seedlings were grown as previously described (Rasheed et al. 2016). Briefly, sterilized seeds were sown on MS medium at 22°C with a 16-h light/8-h dark cycle (60–80 μmol photons m^−2^ s^−1^) for 10 d. The seedlings were transferred to ceramic-based granular soil in pots (Sakatanotane, Yokohama, Japan) and grown for 5 d at 22°C (16-h light/8-h dark cycle, ∼100 μmol m^−2^ s^−1^ photon flux density). The 17-day-old plants were treated with 10 mM ethanol as described earlier. The plants were then subjected to drought stress by removing excess water. For the microarray analysis, the roots and shoots were harvested separately at 0, 3, 24 and 72 h after the ethanol treatment and at 1, 3 and 5 DAD. For the first RNA-seq analysis, shoot samples were collected at 0, 1, 3, 5 and 7 DAD. For the second RNA-seq analysis, the weight of each pot was adjusted by adding water before the onset of drought stress, and then shoot samples were collected at 0, 10 and 13 DAD (**Supplementary Fig. S18**). For the GC–MS and NMR analyses, samples were collected at 24 and 72 h after the ethanol treatment and at 1, 3 and 5 DAD. The harvested samples were immediately placed in liquid nitrogen and stored at −80°C. For the NMR analysis, plants were grown as described earlier, but they were treated with 10 mM ^13^C ethanol. To record the leaf temperature, 19-day-old plants were treated with 10 mM ethanol and then analyzed using the InfReC infrared camera (Nippon Avionics, Yokohama, Japan). Data were analyzed using the InfReC Analyzer NS9500 Lite program. Four leaves per plant were examined to calculate the average temperature of each plant. The stomatal aperture was measured as previously described (Aoki et al. 2019).

Plants were also grown in RIPPS to monitor growth and assess the effect of the ethanol treatment on drought tolerance. Seeds were germinated, and then the seedlings were grown for 9 d in soil-filled 96-well plates after an 8-d stratification process at 4°C in darkness as previously described (Fujita et al. 2018). Plants were then transferred to pots filled with ceramics-based soil and then acclimatized for 5 d. Plants were then treated with ethanol for 3 d by adding 28 ml of 10 mM ethanol solution to the trays containing the pots in two batches. Plants were then subjected to drought stress for 12 d and then rewatered. The soil water content of each pot was adjusted to 1.25 g water/g dry soil just before the initiation of drought treatment. Data were recorded as previously described (Fujita et al. 2018). For the *coi1*-16B experiments, leaf areas were estimated by OpenCV (version 4.0.1) on Python 3.8.5. For prolonged ethanol and acetic acid treatments, 15-day-old *A. thaliana* (Col-0) plants were treated with 10 mM ethanol or acetic acid solutions for 5 weeks. The ethanol and acetic acid solutions were renewed weekly, and data were recorded after the cessation of watering. For examining the effect of acetic acid on seed germination and plant growth, *A. thaliana* (Col-0) seeds were grown on Petri plates containing 1 × MS, 1% sucrose and 0.8% agar medium along with 0, 1, 5 and 10 mM acetic acid. Petri plates containing 30 *A. thaliana* seeds per plate were placed in a growth incubator for 14 d at 22℃ under a 18-h light/6-h dark condition.

Rice (*Oryza sativa* L.; cultivar Nipponbare) seeds were imbibed for 2 d at 30°C and then sown on soil for 19 d (14-h light/10-h dark). The seedlings were subsequently treated with 100 mM ethanol by replacing the water with ethanol solution and then kept for 5 d. Water was then removed, and the plants were rewatered after 4 d. Data were recorded at 21 d after rewatering. Wheat (*Triticum aestivum* L.; cultivar Chinese Spring) seedlings were grown at 22°C (16-h light/8-h dark) for 20 d. The plants were then treated with 50 mM ethanol or water for 3 d as described earlier and subsequently subjected to drought stress. Data were collected at 1 month after rewatering.

### RNA extraction and transcriptome analysis

We performed microarray and RNA-seq analysis to identify the differentially regulated genes in response to the ethanol treatment. We first performed a microarray analysis to monitor the transcriptomic changes at 3, 24 and 72 h after the ethanol treatment and at 1, 3 and 5 DAD in the root and shoot tissues in Arabidopsis (**Supplementary Fig. S18A**). We then performed the RNA-seq analysis to monitor the transcriptomic changes specifically during the later stages of drought stress, i.e. after 7 DAD in the shoot tissues (**Supplementary Fig. S18B**). Third transcriptomic analysis (second RNA-seq analysis) was performed to monitor the transcriptomic changes in the shoot tissues (**Supplementary Fig. S18B**) with modified growth conditions to correlate our data with phenotyping analyses (RIPPS). Total RNA was extracted from all biological replicates using the *mir*Vana™ miRNA Isolation Kit (Thermo Fisher Scientific, Waltham, MA, USA) according to the manufacturer’s instructions.

Microarray analyses were performed as previously described (Rasheed et al. 2016). Briefly, fluorescent-labeled (Cy3) complementary DNA was prepared from 400 ng of total RNA for each sample using the Quick Amp Labeling kit (Agilent Technologies, Santa Clara, CA, USA) and then hybridized to an Agilent Arabidopsis custom microarray (GPL19830). Three biological replicates were processed per treatment. Arrays were scanned using the G2505B microarray scanner (Agilent Technologies), and the data were processed and analyzed using Greenspring GX (v.14.9; Agilent Technologies) with quantile normalization as previously described (Takaoka et al. 2018). Statistical significance was assessed according to a one-way analysis of variance with Benjamini-Hochberg correction and a 95% confidence interval (corrected *P* < 0.1). Genes with a false discovery rate (FDR) value less than 0.1 and at least a 2-fold change in expression were regarded as differentially expressed.

For the RNA-seq analysis, the sequencing library was prepared using the Lasy-Seq method (Kamitani et al. 2019). Specifically, 200 ng of total RNA was used per sample. The library was sequenced using the 151-bp paired-end mode of the HiSeq X Ten (Illumina, San Diego, CA, USA). RNA-seq analyses were performed with R1 reads. Low-quality reads and adapters were trimmed using Trimmomatic version 0.39 (http://www.usadellab.org/cms/?page=trimmomatic) with ‘ILLUMINACLIP:TruSeaq3-SE.fa:2:30:10 LEADING:3 TRAILING:3 SLIDINGWINDOW:4:15 MINLEN:36’ options. HISAT2 (http://daehwankimlab.github.io/hisat2/) version 2.2.1 was used to map the reads to the *A. thaliana* reference genome (TAIR10) with the ‘--max-intronlen 5000’ option. Aligned reads within gene models were counted using featureCounts version 2.0.1 (http://subread.sourceforge.net/) with the ‘--fracOverlap 0.5 -O -t gene -g ID -s 1 --primary’ options. Differentially expressed genes were identified using R version 4.0.4 (https://www.r-project.org/) and DESeq2 version 1.30.1 (https://bioconductor.org/packages/release/bioc/html/DESeq2.html) package. Genes with an FDR <0.1 in each comparison were identified as differentially expressed genes. The GO analysis was performed using an online program (http://www.pantherdb.org). The microarray and first and second RNA-seq data have been deposited into the GenBank database with the accession numbers GSE201609, GSE201379 and GSE201380, respectively.

Additionally, qRT-PCR analyses were performed to confirm the changes in gene expression under progressive drought stress as well as to access the reliability of the microarray and RNA-seq analyses. Details regarding the qRT-PCR primers are provided in **Supplementary Table S12**.

### ABA measurement in the roots and shoots

Plants were grown, and samples were collected as described earlier. Endogenous ABA was extracted from the roots and shoots separately using 80% (v/v) acetonitrile containing 1% (v/v) acetic acid. The hormone contents were determined using a ultra performance liquid chromatography (UPLC)–MS/MS system consisting of a quadrupole/time-of-flight (TOF) tandem mass spectrometer (Triple TOF 5600; SCIEX, Concord, ON, Canada) and a Nexera UPLC system (Shimadzu Corp., Kyoto, Japan) as previously described (Kanno et al. 2016).

### Metabolite profiling

Metabolite profiling was performed as previously described (Kusano et al. 2007, Sugi et al. 2021), with slight modifications. Briefly, extracts were prepared by adding 1 ml of extraction medium containing 10 stable isotope reference compounds to ∼25 mg of roots or shoots (fresh weight). After centrifuging the extract, a 200-μl aliquot of the supernatant was collected and dried via evaporation and used for the derivatization step as previously described (Kusano et al. 2007). The derivatized samples were analyzed by GC and TOF MS (GC–TOF-MS) (LECO, St Joseph, MI, USA). Data were normalized using the cross-contribution compensating multiple standard normalization algorithm (Redestig et al. 2009).

The relative quantification of glucosinolates was performed using an LC–MS/MS system as previously reported (Sawada et al. 2017), with some modifications (Sugiyama et al. 2021). After extracting samples (4 mg/ml), 50 μl aliquots were dried for 1 h and then dissolved in 500 μl of water for a concentration of 0.4 mg/ml. Next, a 90 μl aliquot of the extract was mixed with 10 μl of 10 μM sinigrin as the internal standard.

### ^13^C-metabolic tracing experiments by two-dimensional NMR spectroscopy

Labeled ethanol (^13^CH_3_^13^CH_2_OH; Cambridge Isotope Laboratories, Tewksbury, MA, USA) was used for the NMR analysis during the ^13^C-metabolite tracing experiments that were performed via root uptake (Kikuchi et al. 2004). Lyophilized samples were ground to a fine powder, and then 10 mg of powdered material per sample was dissolved in 600 µl of 0.1 M potassium phosphate buffer (pH 7). The samples were incubated at 65°C for 15 min and processed as previously described (Sekiyama et al. 2011). Two-dimensional ^1^H-^13^C heteronuclear single quantum coherence (HSQC) spectra were recorded as previously described (Chikayama et al. 2010) using a 700-MHz NMR spectrometer (Bruker BioSpin, Rheinstetten, Germany). The same samples were also analyzed by zero-quantum filtered total correlation spectroscopy (ZQF-TOCSY) according to a published method (Komatsu et al. 2016). To normalize the signal intensities for the ^1^H-^13^C HSQC and ZQF-TOCSY spectra, they were standardized according to the 3-(trimethylsilyl)-1-propanesulfonic acid sodium salt intensity. The peak intensity was normalized against the unit vector length for each peak.

## Supplementary Material

pcac114_Supp

## Data Availability

The data presented in this article are available in Gene Expression Omnibus at https://www.ncbi.nlm.nih.gov/geo/ and can be accessed with GSE201609, GSE201379 and GSE201380.
