# Supplementary material for: Ethanol-Mediated Novel Survival Strategy against Drought Stress in Plants
Source: Plant Cell Physiol. 2022 Aug 25;63(9):1181–92. doi: 10.1093/pcp/pcac114 (PMC9474946; doi:10.1093/pcp/pcac114)
Supplement: pcac114_Supp [file pcac114_supp.zip › pcp-2022-e-00145-File009.pdf]

Ethanol-mediated novel survival strategy against drought stress in plants

Khurram Bashir, Daisuke Todaka, Sultana Rasheed, Akihiro Matsui, Zarnab Ahmad, Kaori Sako, Yoshinori Utsumi, Vu Anh Thu, Maho Tanaka, Satoshi Takahashi, Junko Ishida, Yuuri Tsuboi, Shunsuke Watanabe, Yuri Kanno, Eigo Ando, Kwang-Chul Shin, Makoto Seito, Hinata Motegi, Muneo Sato, Rui Li, Saya Kikuchi, Miki Fujita, Miyako Kusano, Makoto Kobayashi, Yoshiki Habu, Atsushi J. Nagano, Kanako Kawaura, Jun Kikuchi, Kazuki Saito, Masami Yokota Hirai, Mitsunori Seo, Kazuo Shinozaki, Toshinori Kinoshita, and Motoaki Seki*****

***Corresponding author**:
Motoaki Seki
TEL: +81-(0)45-503-9587
FAX: +81-(0)45-503-9584
Email: motoaki.seki@riken.jp

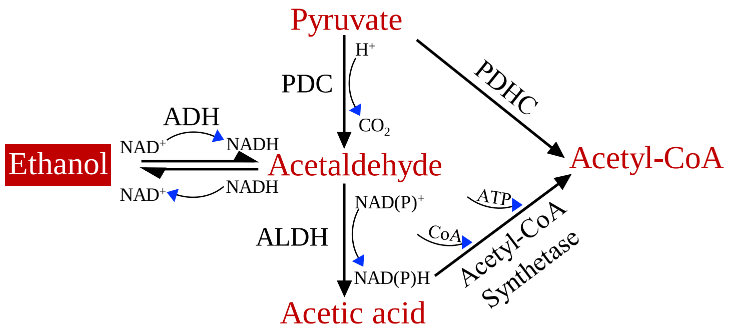

Supplementary Fig. S1. Ethanol and acetic acid biosynthesis pathway in plants. Pyruvate is converted into Acetyl-CoA by pyruvate dehydrogenase complex (PDHC). Under anaerobic and stress conditions, pyruvate is converted into acetaldehyde by PDC. Acetaldehyde could be converted into ethanol. The conversion of acetaldehyde into ethanol is a reversible process. Acetaldehyde could also be converted into acetic acid. Three genes (*ALDH2B4*, *ALDH2B7*, and *ALDH2C4*) are suggested to be involved in the conversion of acetaldehyde into acetic acid.

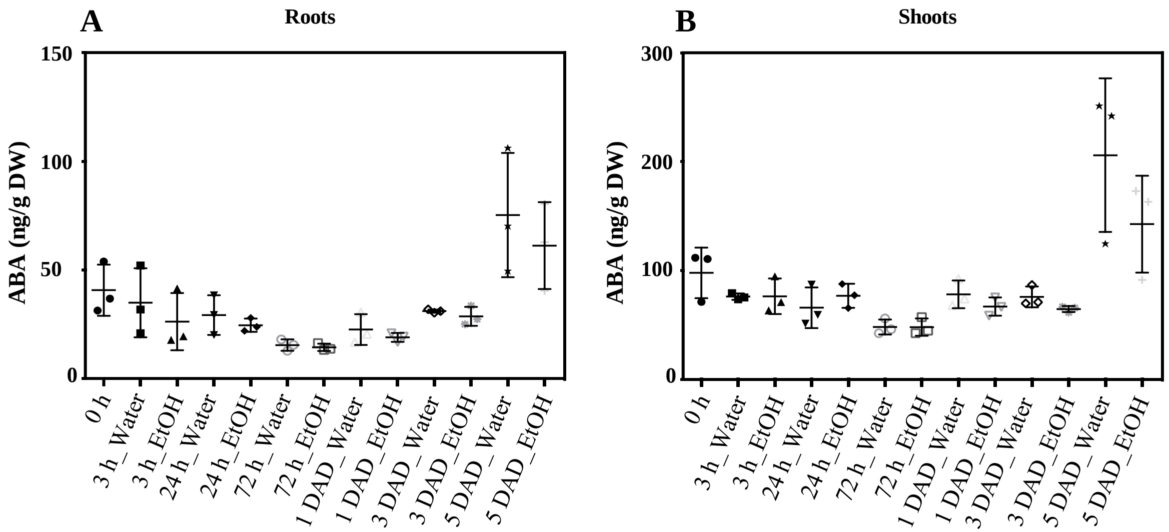

Supplementary Fig. S2. Ethanol treatment does not change the levels of endogenous ABA.
Two-week-old *Arabidopsis* plants were treated with 10 mM ethanol or water for 3-days and then subjected to drought stress treatment. **A**: ABA contents in roots. **B**: ABA contents in shoots. Samples were collected before the application of ethanol (0 h) and 3, 24, and 72 h after the ethanol treatment, and 1, 3, and 5 days after the drought (DAD) stress treatment. No statistical differences were observed according to the t-test ($p < 0.05$; n = 3).

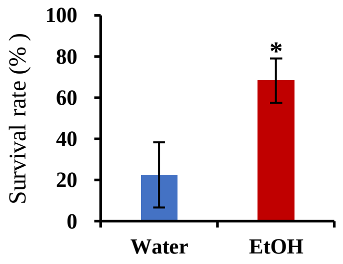

Supplementary Fig. S3. Ethanol-mediated drought stress tolerance in Landsberg *erecta* ecotype. Two-week-old WT *Arabidopsis* plants (ecotype: Landsberg *erecta*) were treated with 10 mM ethanol for 3-days and then subjected to drought stress treatment. Column bars followed by asterisks are statistically different from the control according to the SNK test ($p < 0.05$; $n = 3$).

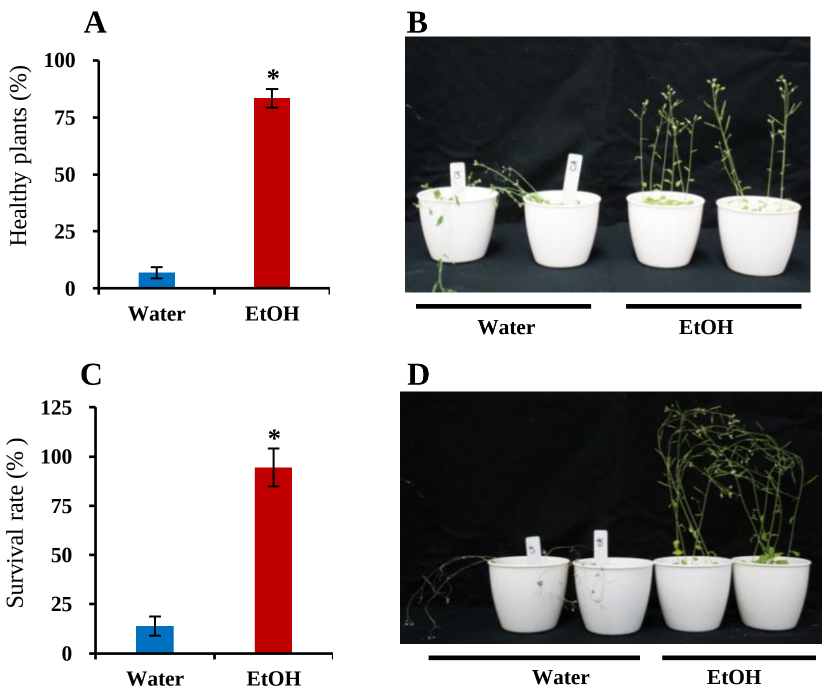

Supplementary Fig. S4. Ethanol enhances drought stress tolerance in ceramics granular soil. Ten-days old *Arabidopsis* plants were transferred to ceramics granular soil, acclimatized for 5-days, treated with 10 mM ethanol (EtOH) for 3-days, and then subjected to drought stress treatment. Plants treated with water were used as the control. **A**-**B**: Percentage of healthy plants after 9-day drought stress treatment (before rewatering). **C**-**D**: Survival rate recorded at 3 days after rewatering. Column bars followed by asterisks are statistically different from control according to the SNK test ($p < 0.05$; $n = 4$).

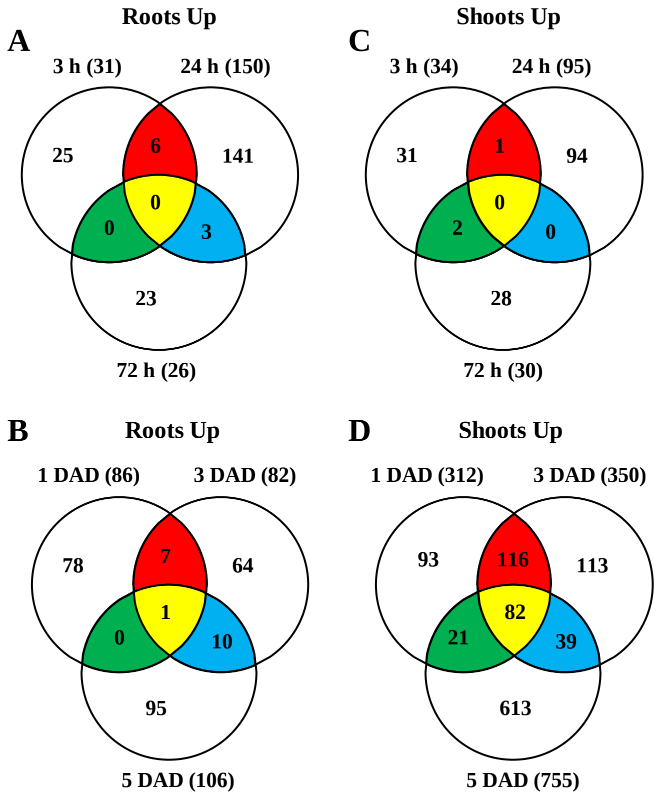

Supplementary Fig. S5. Venn diagram representing the transcriptomic changes in response to ethanol treatment followed by drought stress treatment.

Two-week-old *Arabidopsis* plants were treated with 10 mM ethanol or water for 3-days and then subjected to drought stress treatment. Genes upregulated at 3, 24, and 72 h after ethanol treatment in roots (**A**) and shoots (**C**) and 1, 3, and 5 days after drought (DAD) stress treatment in roots (**B**) and shoots (**D**), n = 3, fold change ≥ 2; FDR < 0.1.

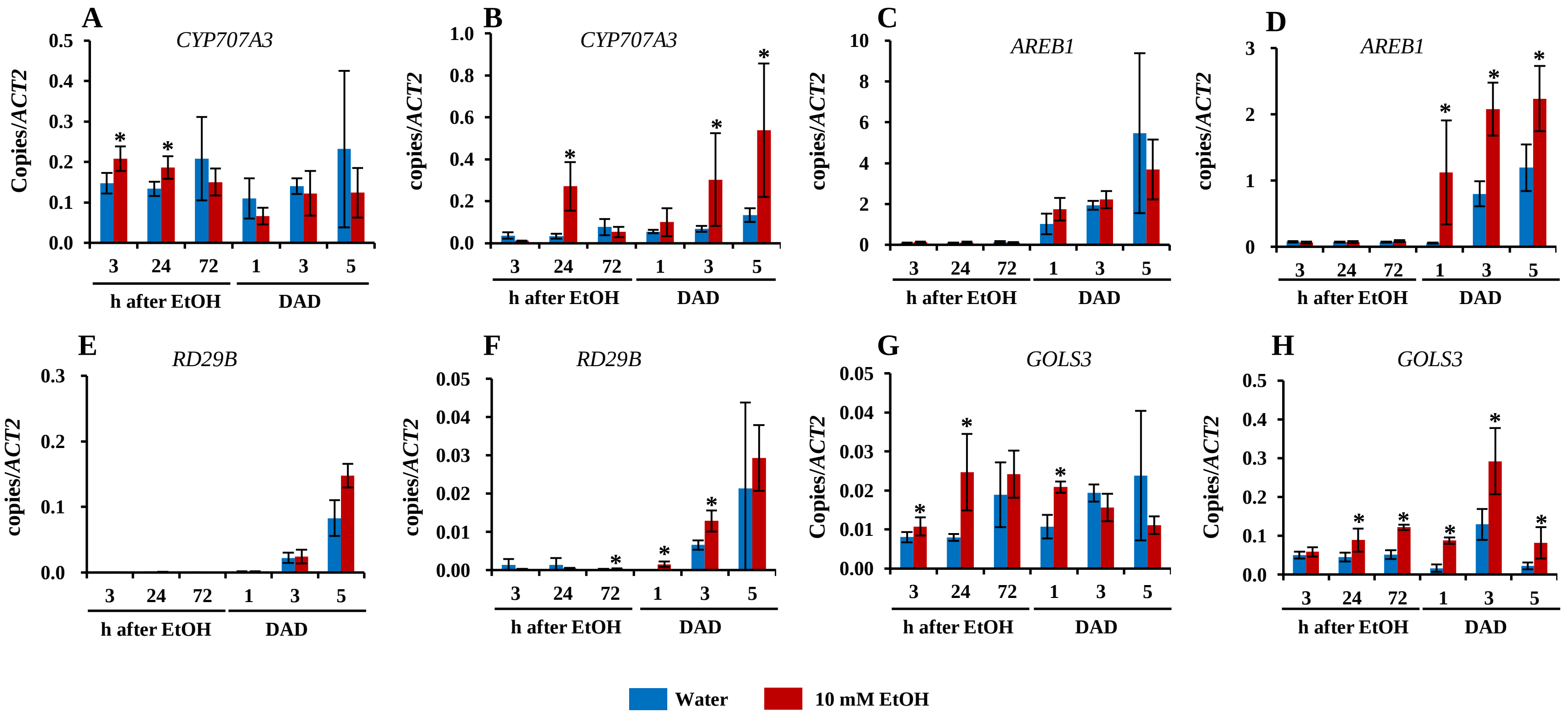

Supplementary Fig. S6. qRT-PCR analysis to confirm the microarray results.
qRT-PCR analyses were performed to confirm the results obtained through microarray analysis and are shown as copies per *Actin2 (ACT2)* at different time points. Samples were collected at 3, 24, and 72 h after ethanol treatment and then 1, 3, and 5-days after drought (DAD) stress treatment. Changes in the expression of **A-B**: *CYP707A3* in roots (**A**) and shoots (**B**). **C-D**: *AREB1* in roots (**C**) and shoots (**D**). **E-F**: *RD29B* in roots (E) and shoots (**F**). **G-H**: *GOLS3* in roots **(G)** and shoots (**H**). Column bars followed by asterisks are statistically different from the control according to the t-test ($p < 0.05$; n = 3).

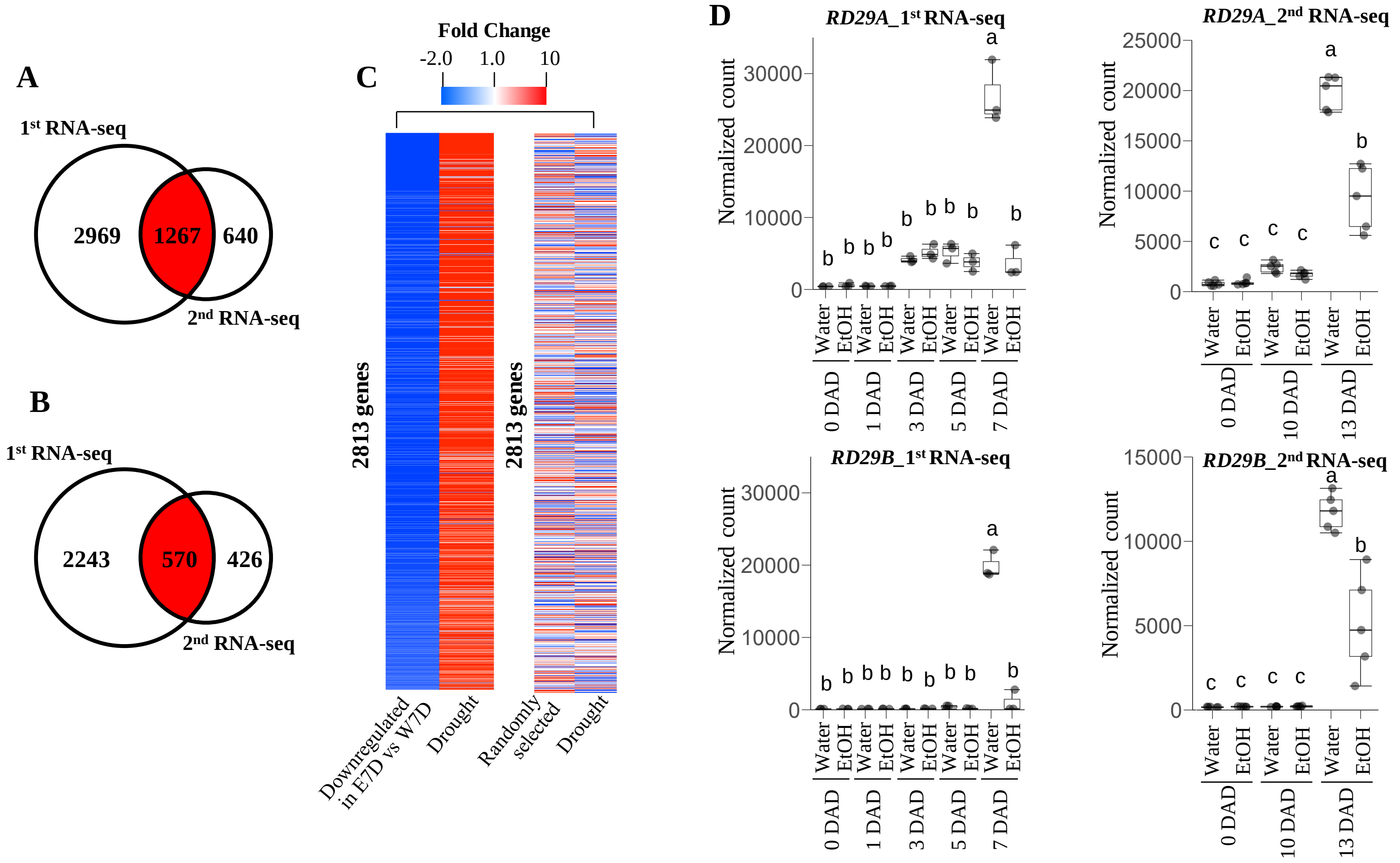

Supplementary Fig. S7. Summary of RNA-seq analyses.
A-B: Genes up **(A)** and down **(B)** -regulated in RNA-seq analyses. The data of 7 DAD and 13 DAD were used for the analysis of 1st RNA-seq and 2nd RNA-seq, respectively. Independent sampling and RNA extraction were performed for both RNA-seq analysis, **C:** Heatmap analysis of 2,813 genes downregulated at ethanol-treated 7DAD compared with water-treated 7DAD in 1st RNA-seq. Randomly selected 2,813 genes were chosen from all the *Arabidopsis* genes. The drought responsiveness in the right tiles is based on the results of 1st RNA-seq (water-treated 7 DAD vs water-treated 0 DAD). **D:** Normalized counts of *RD29A* and *RD29B* during drought stress treatment. Different letters indicate significant differences analyzed by Tukey-Kramer multiple comparison test ($p < 0.05$).

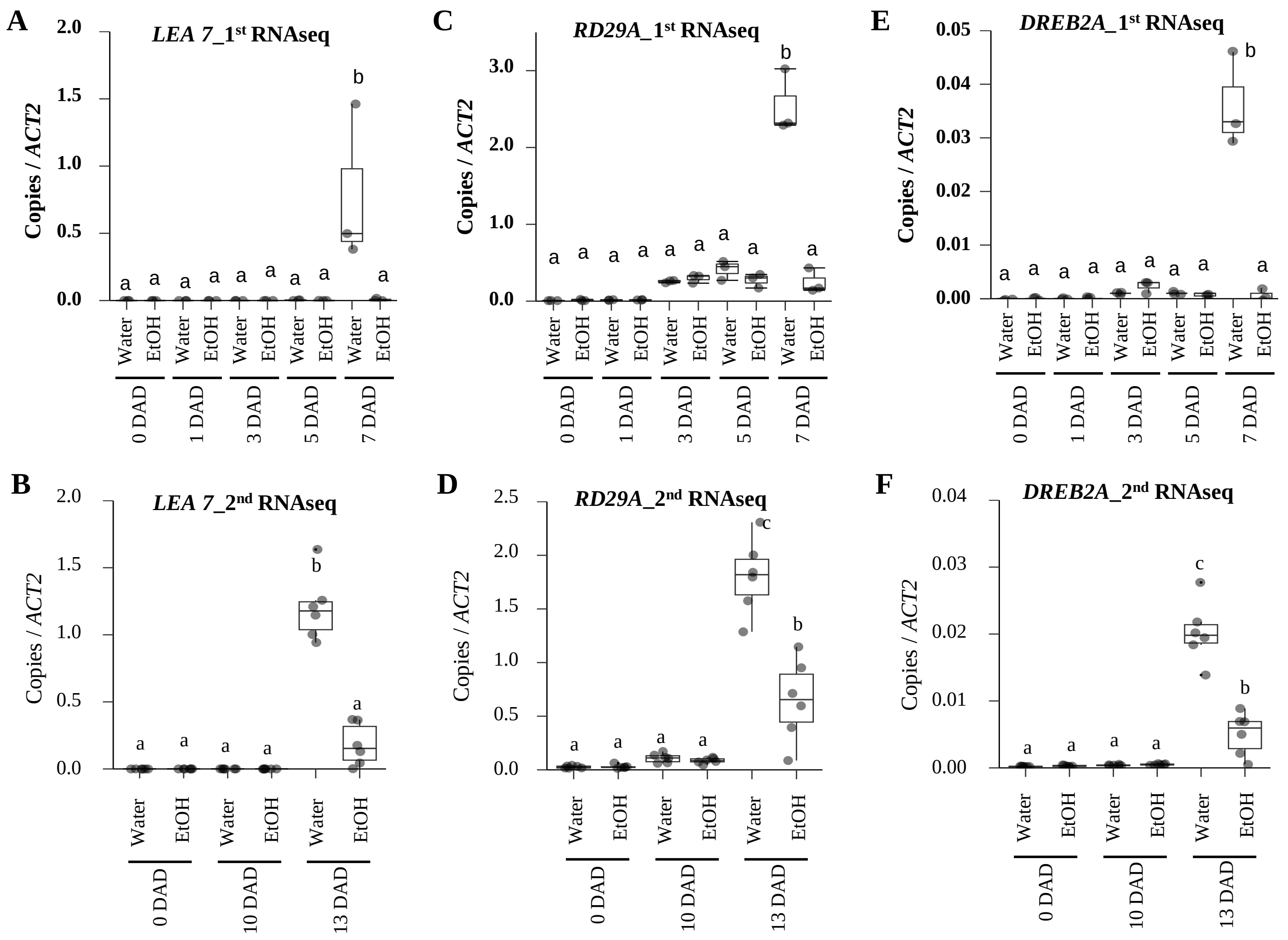

Supplementary Fig. S8. qRT-PCR analysis to confirm the RNA-seq results.

RNA-seq results were validated by qRT-PCR analyses. Data are presented as copies per *Actin2* (*ACT2*) at different time points. Samples were collected at 0, 1, 3, 5, and 7 DAD in the 1st RNAseq (**A**, **C**, and **E**) and 0, 10, and 13 DAD in the 2nd RNA-seq (**B**, **D**, and **F**). Changes in the expression of *late embryogenesis abundant 7* (*LEA 7*; **A** and **B**), *RD29A* (**C** and **D**), and *DREB2A* (**E** and **F**). Different letters indicate significant differences according to Tukey's test ($p < 0.05$; $n = 3$ to 6).

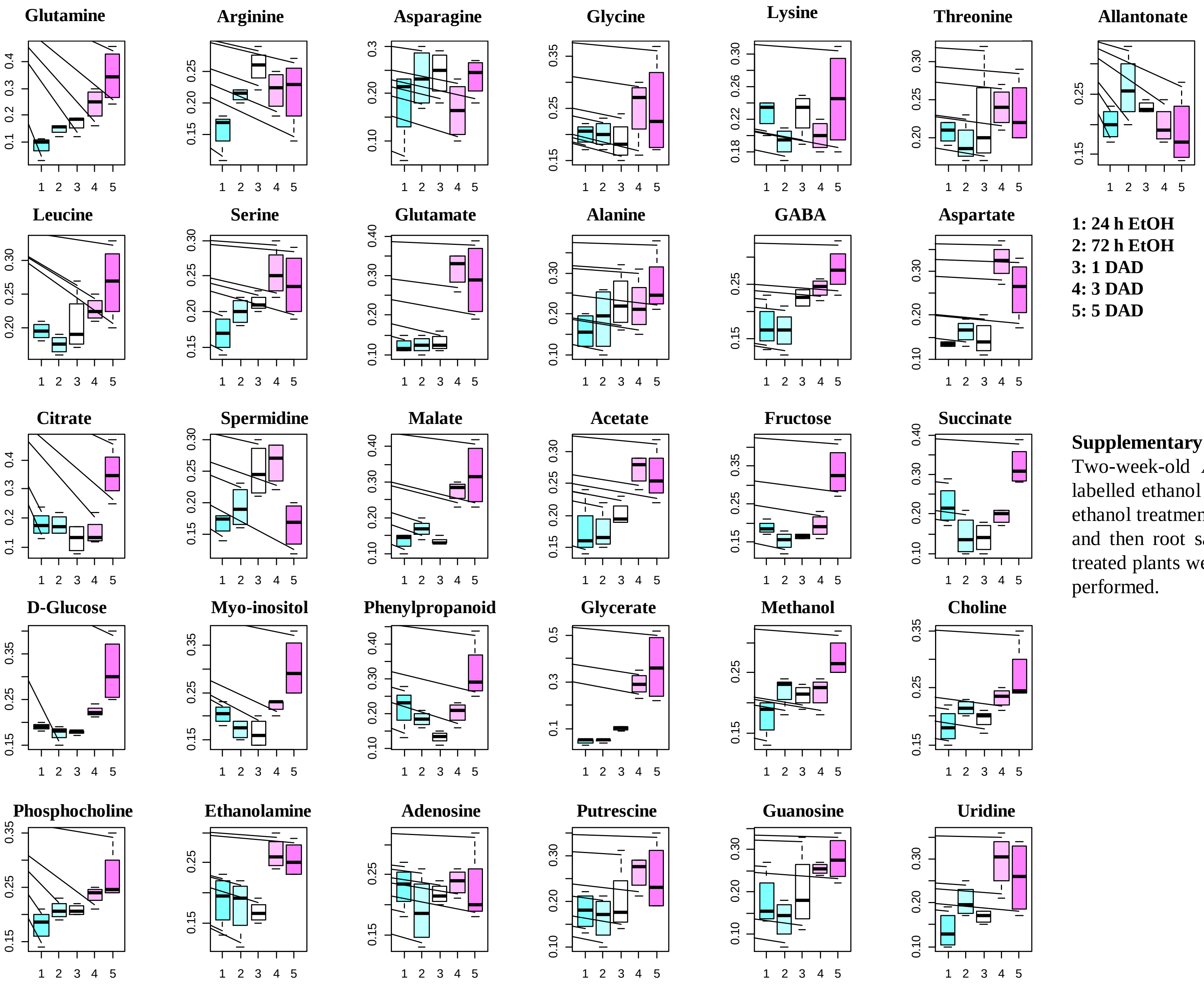

Supplementary Fig. S9. Summary of root HSQC-NMR analysis. Two-week-old *Arabidopsis* plants were treated with 10 mM ^{13}C labelled ethanol and root samples were collected at 24 and 72 h after ethanol treatment. At this point, drought stress treatment was initiated and then root samples were collected at 1, 3, and 5 DAD. Water-treated plants were not analyzed and thus statistical analyses were not performed.

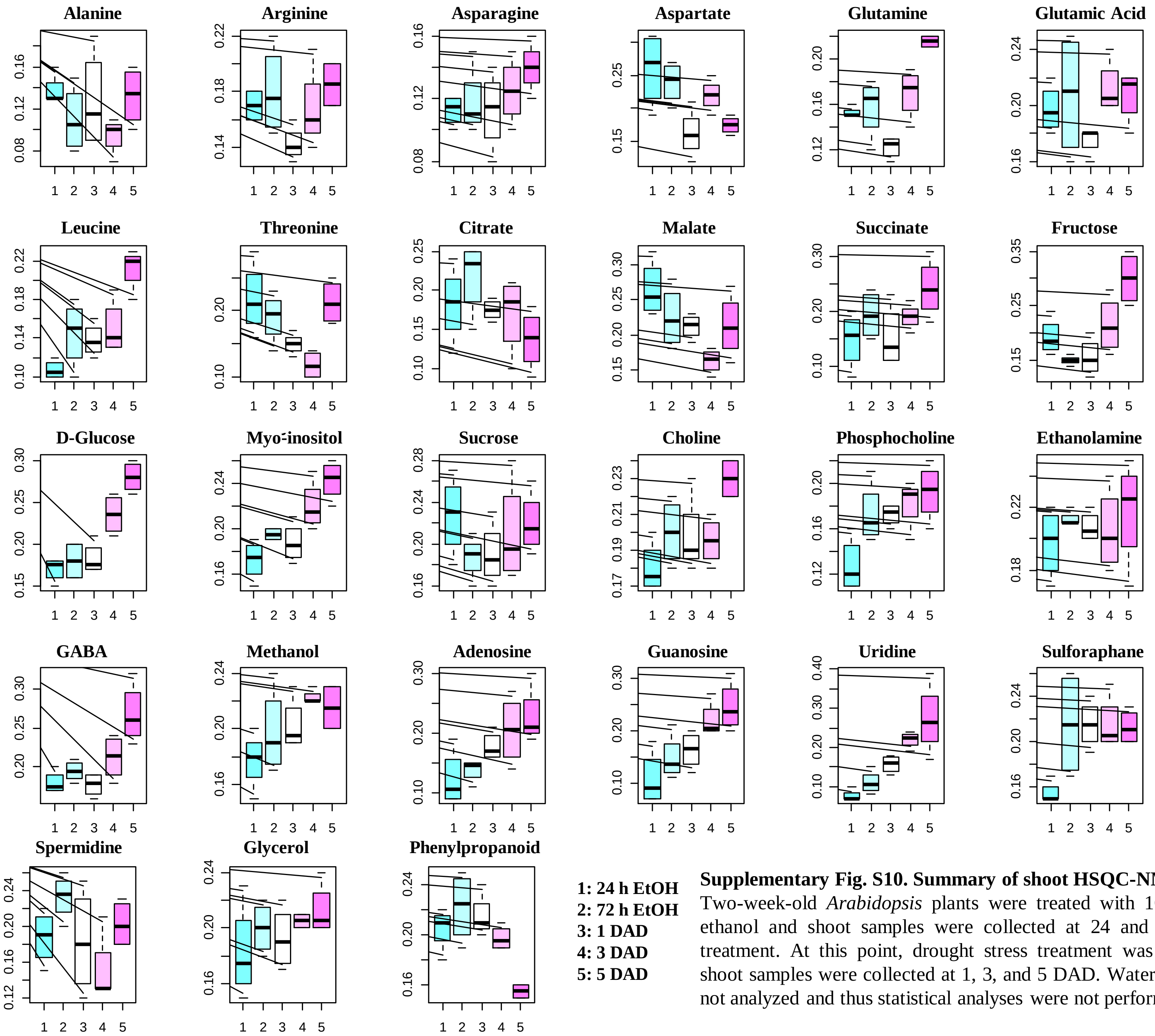

Supplementary Fig. S10. Summary of shoot HSQC-NMR analysis. Two-week-old *Arabidopsis* plants were treated with 10 mM ^{13}C labelled ethanol and shoot samples were collected at 24 and 72 h after ethanol treatment. At this point, drought stress treatment was initiated, and then shoot samples were collected at 1, 3, and 5 DAD. Water-treated plants were not analyzed and thus statistical analyses were not performed.

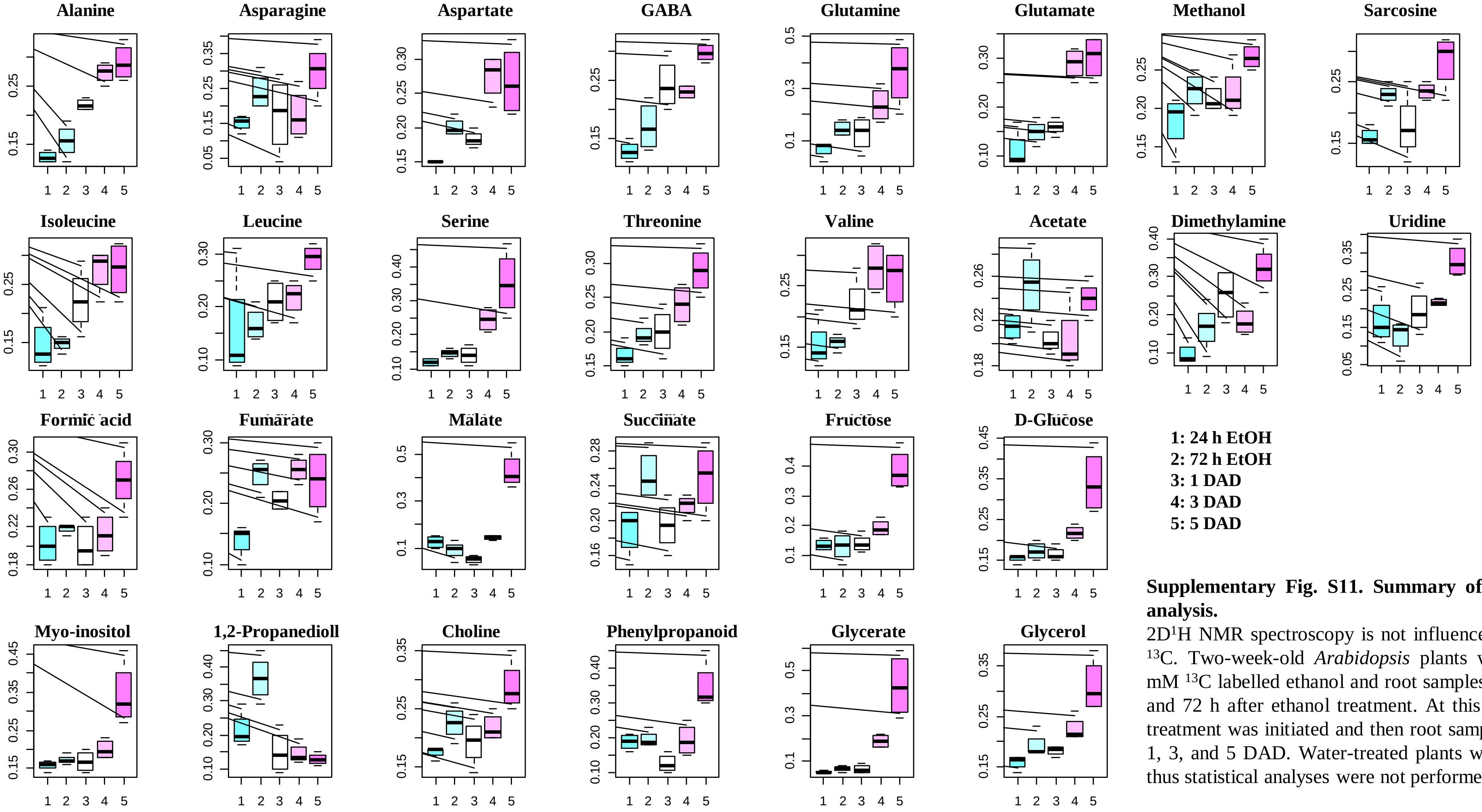

Supplementary Fig. S11. Summary of root 2D-*jres* NMR analysis.
2D^{1}H NMR spectroscopy is not influenced by the presence of ^{13}C. Two-week-old *Arabidopsis* plants were treated with 10 mM ^{13}C labelled ethanol and root samples were collected at 24 and 72 h after ethanol treatment. At this point, drought stress treatment was initiated and then root samples were collected at 1, 3, and 5 DAD. Water-treated plants were not analyzed and thus statistical analyses were not performed.

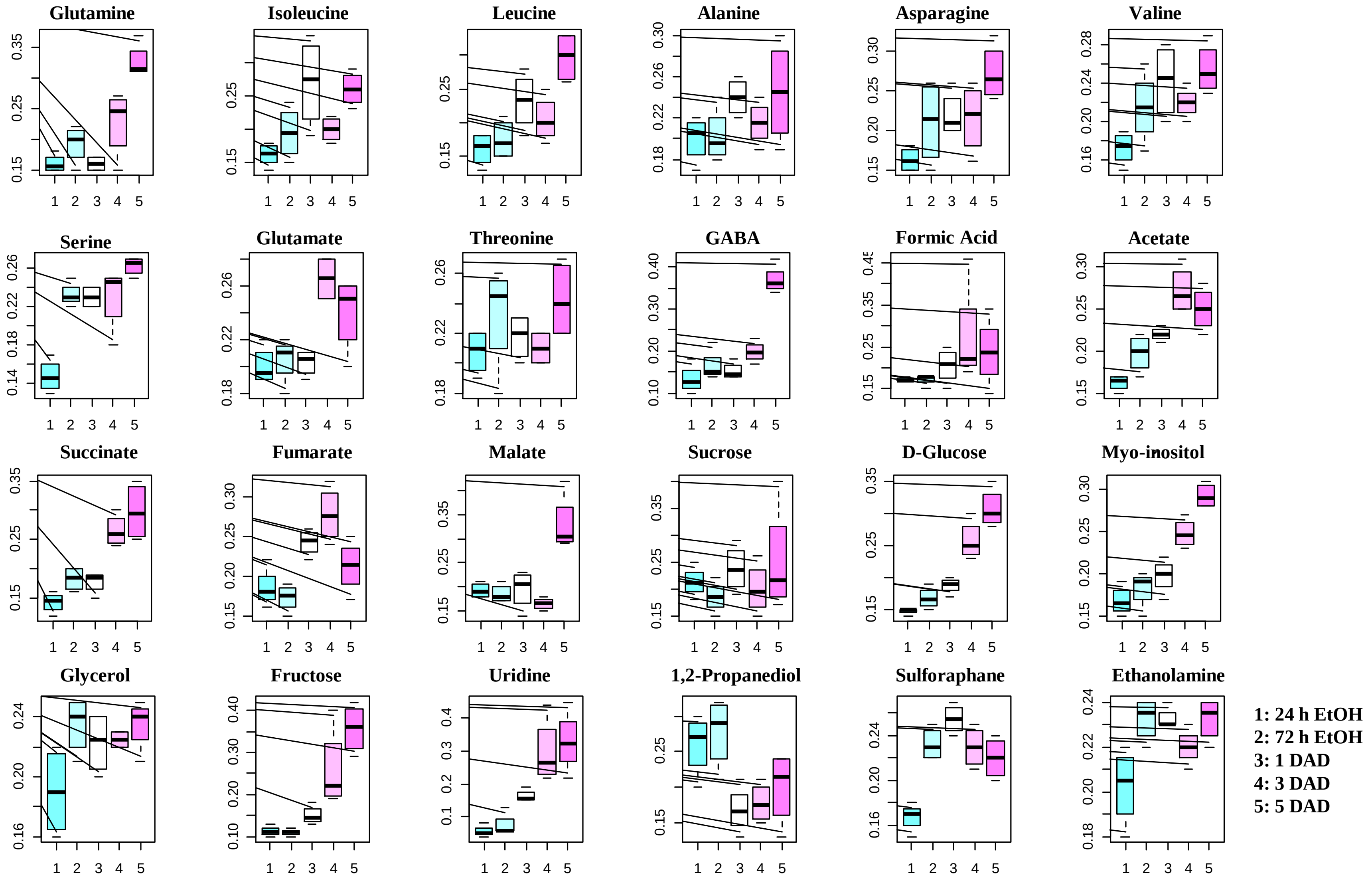

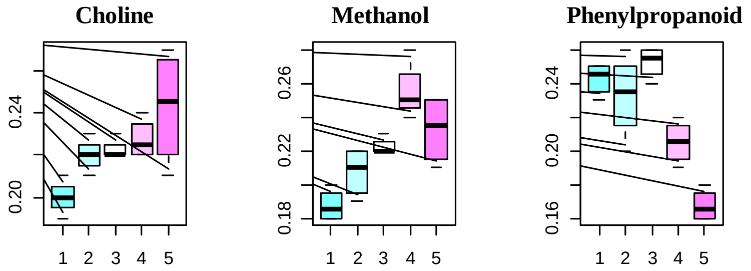

Supplementary Fig. S12. Summary of shoot 2D-jres NMR analysis.
2D ^{1}H NMR spectroscopy is not influenced by the presence of ^{13}C. Two-week-old *Arabidopsis* plants were treated with 10 mM ^{13}C labelled ethanol and shoot samples were collected at 24 and 72 h after ethanol treatment. At this point drought stress treatment was initiated and then shoot samples were collected at 1, 3, and 5 DAD. Water-treated plants were not analyzed and thus statistical analyses were not performed.

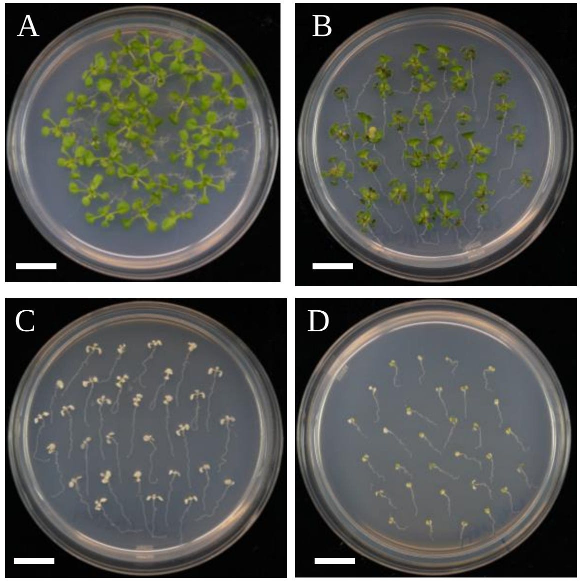

Supplementary Fig. S13. Effect of acetic acid on seed germination and seedling growth in *Arabidopsis*.
Photos of *Arabidopsis* WT plants grown on MS media containing 0 (**A**), 1 (**B**), 5 (**C**), and 10 (**D**) mM acetic acid for 2 weeks.
Scale bar = 1cm

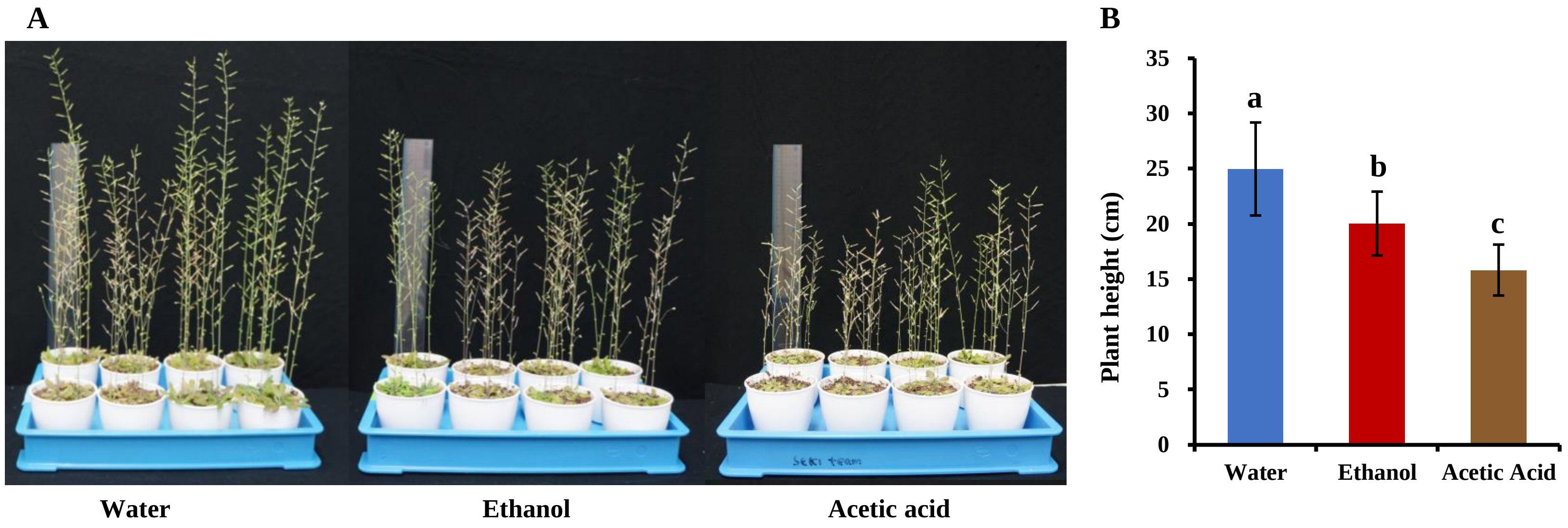

Supplementary Fig. S14. Continuous supply of ethanol and acetic acid affects plant growth.
15-days old *Arabidopsis* plants were treated with water, 10 mM ethanol, or 10 mM acetic acid for 5 weeks. **A**: Photograph of water, ethanol, and acetic acid-treated plants. **B**: Difference among the plant height of water, ethanol, and acetic acid-treated plants. Column bars followed by different letters are statistically different from each other according to the SNK test ($p < 0.05$; $n = 8$).

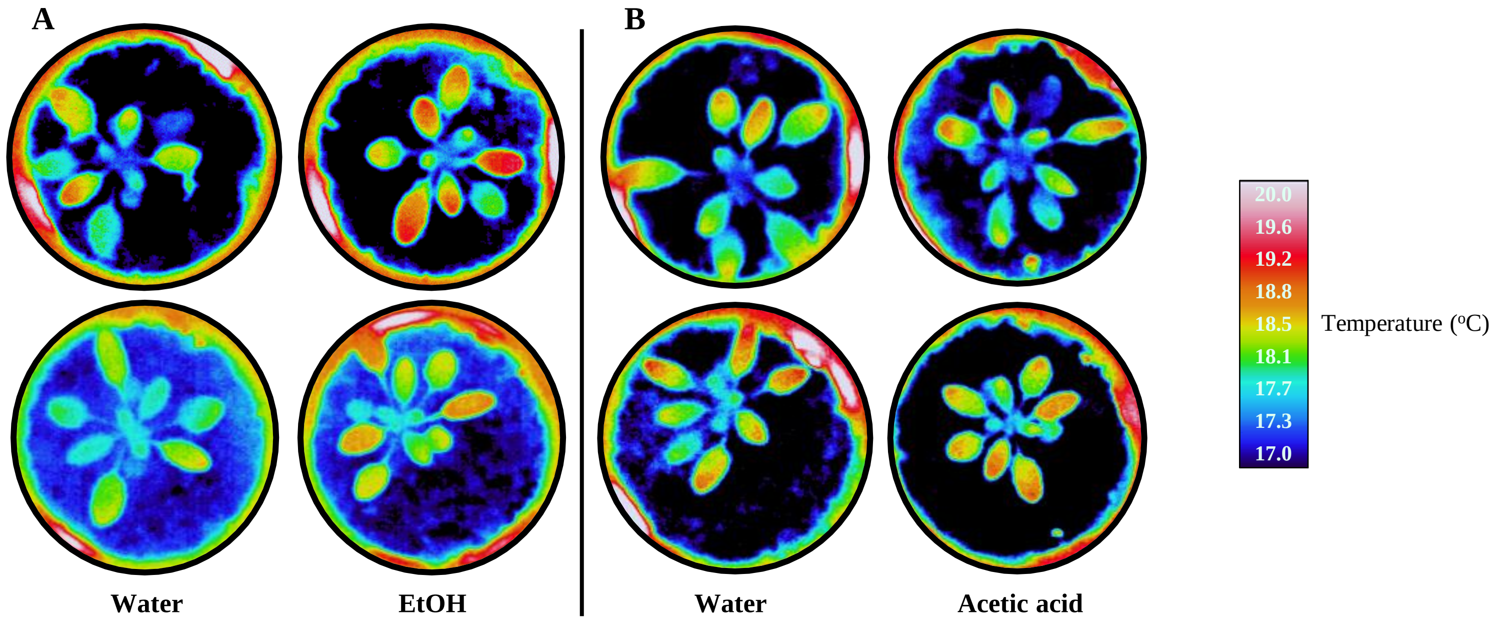

Supplementary Fig. S15. Acetic acid treatment does not increase leaf temperature.
Two-week-old *Arabidopsis* plants were treated with 10 mM ethanol or 10 mM acetic acid. Ethanol treatment increases leaf temperature (**A**) while the acetic acid treatment does not increase leaf temperature (**B**). Leaf temperature was measured at 72 h after the ethanol or acetic acid treatment in two independent experiments.

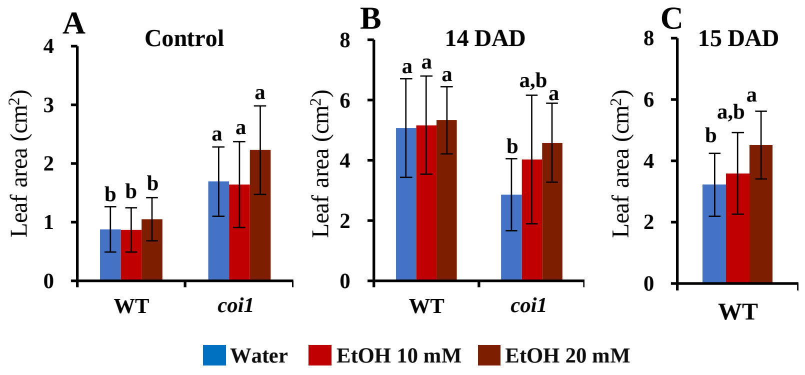

Supplementary Fig. S16. Ethanol response is similar between WT and *coi1* mutant (*coi1-16*).

Three-week-old WT (Col-0) and *coi1* mutant **(*coi1-16*)** plants were treated with 0, 10, and 20 mM ethanol for 3 days and then subjected to drought stress. Leaf area (cm^2) was measured at **A**: before the ethanol treatment**, B:** 14 days after drought (14 DAD), and **C:** 15 DAD. Column bars followed by different letters are statistically different from each other according to the SNK test ($p < 0.05$; n = 16).

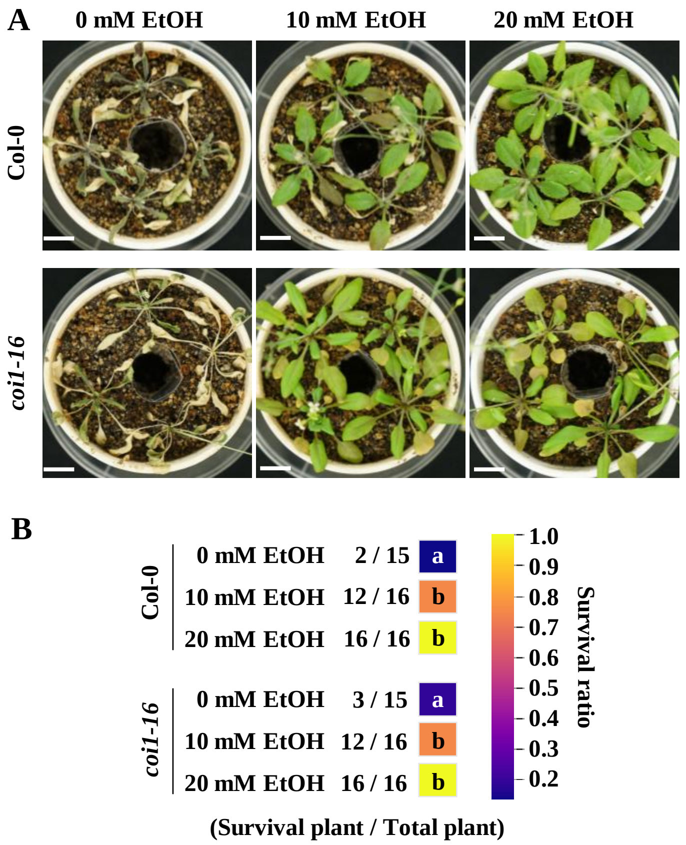

Supplemental Figure S17. Ethanol enhances drought tolerance in *coi1* mutant plants.
Three-week-old WT (Col-0) and *coi-1* (*coi1-16*) mutant plants were treated with 0, 10, and 20 mM EtOH for 3 days. Drought treatment was performed for 16 days in Col-0 and 14 days in *coi1-16*. **A**: Photographs were taken after rewatering for 4 days in Col-0 and 5 days in *coi1-16*. Scale bar = 1cm. **B:** The survival ratio of WT and *coi1-16* plants. Color tiles followed by different letters are statistically different from each other according to Pairwise comparisons using Fisher's exact test ($p < 0.05$; $n = 16$). P values were adjusted by the method of holm. In this experiment, n shows the number of treated plants.

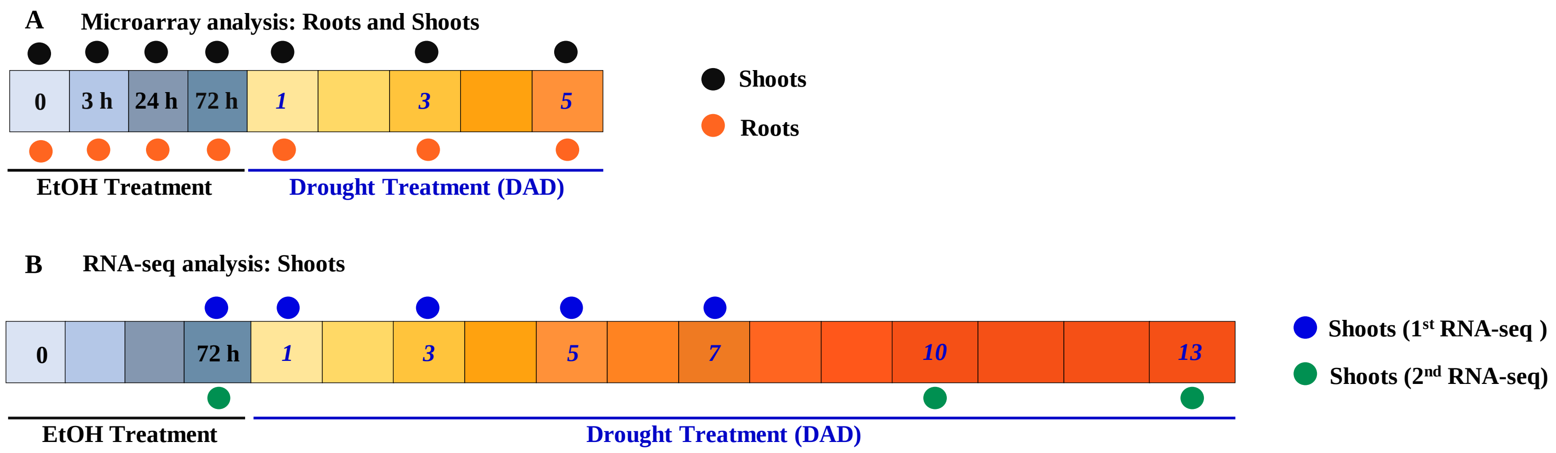

Supplementary Fig. S18. Sampling time points for the transcriptomic analysis.
A: Sampling time points of shoots and roots for the microarray analysis are shown as circles of black and orange, respectively.
B: Sampling time points of shoots for the 1st and 2nd RNA-seq analysis are shown as circles of blue and green, respectively. 72 h and 0 DAD represent the same time point.
